# Microbial metabolomic responses to changes in temperature and salinity along the western Antarctic Peninsula

**DOI:** 10.1038/s41396-023-01475-0

**Published:** 2023-09-15

**Authors:** H. M. Dawson, E. Connors, N. G. Erazo, J. S. Sacks, V. Mierzejewski, S. M. Rundell, L. T. Carlson, J. W. Deming, A. E. Ingalls, J. S. Bowman, J. N. Young

**Affiliations:** 1 School of Oceanography, University of Washington, Seattle, WA 98195 USA; 2 Scripps Institution of Oceanography, UC San Diego, La Jolla, CA 92037 USA; 3 Center for Marine Biodiversity and Conservation, UC San Diego, La Jolla, CA 92037 USA; 4 School of Earth and Space Exploration, Arizona State University, Tempe, AZ 85287 USA; 5 Center for Microbiome Innovation, UC San Diego, La Jolla, CA 92037 USA

**Keywords:** Water microbiology, Biogeochemistry, Climate-change impacts, Environmental chemistry, Climate-change ecology

## Abstract

Seasonal cycles within the marginal ice zones in polar regions include large shifts in temperature and salinity that strongly influence microbial abundance and physiology. However, the combined effects of concurrent temperature and salinity change on microbial community structure and biochemical composition during transitions between seawater and sea ice are not well understood. Coastal marine communities along the western Antarctic Peninsula were sampled and surface seawater was incubated at combinations of temperature and salinity mimicking the formation (cold, salty) and melting (warm, fresh) of sea ice to evaluate how these factors may shape community composition and particulate metabolite pools during seasonal transitions. Bacterial and algal community structures were tightly coupled to each other and distinct across sea-ice, seawater, and sea-ice-meltwater field samples, with unique metabolite profiles in each habitat. During short-term (approximately 10-day) incubations of seawater microbial communities under different temperature and salinity conditions, community compositions changed minimally while metabolite pools shifted greatly, strongly accumulating compatible solutes like proline and glycine betaine under cold and salty conditions. Lower salinities reduced total metabolite concentrations in particulate matter, which may indicate a release of metabolites into the labile dissolved organic matter pool. Low salinity also increased acylcarnitine concentrations in particulate matter, suggesting a potential for fatty acid degradation and reduced nutritional value at the base of the food web during freshening. Our findings have consequences for food web dynamics, microbial interactions, and carbon cycling as polar regions undergo rapid climate change.

## Introduction

Surface ocean temperatures along the western Antarctic Peninsula (WAP) have increased by >1 °C since the 1950s [1]. Associated changes in sea-ice thickness, extent, and duration have also been observed, along with increased occurrence of freshwater melt ponds and under-ice melt lenses [2, 3]. Glacial ice-mass loss has also contributed to greater freshwater inputs into coastal regions [4], with an observed overall warming and freshening of Southern Ocean waters [5]. The largest glacial ice-mass loss in Antarctica was observed along the WAP [6], where a reduction in sea-ice extent and shorter sea-ice season have also been observed [7–10].

With global air temperatures expected to rise at least 1–3 °C over the next century [11], coastal Antarctic ecosystems will likely be exposed to further warming, freshening, and alterations to seasonal ice dynamics. These changes can be expected to alter the temporal and spatial extent of unique microbial habitats that are created and lost seasonally with the formation and melting of sea ice, from and into the surrounding seawater. Microbial community composition is distinct across these polar environments [12–14], but the mechanistic drivers are not fully understood. In terms of biomass, ice-associated communities are usually dominated by sea-ice algae, which can account for 55–65% of coastal Antarctic primary production [15] and provide fixed carbon to microbes and higher trophic levels, particularly during winter when primary production in the water column is nearly zero [16]. Newly formed sea-ice algal communities generally resemble the mixed community in the source seawater, while older ice loses centric diatoms and becomes dominated by large pennate diatoms; spring ice shows increasingly mixed communities before diatoms are lost during ice melt, leaving many flagellates [14]. Prokaryotic taxa are similarly distinct, with newly formed sea-ice communities often dominated by Alphaproteobacteria and Archaea, resembling source seawater, and spring ice shifting to dominance by Gammaproteobacteria and Flavobacteriia [13, 17, 18].

Transitions between polar marine habitats are associated with shifts in light, nutrients, temperature, and salinity [19–22]. However, the combined role of temperature and salinity in shaping community composition among polar microorganisms, and the organic matter they use and produce, has not been fully explored. Limited work in sea ice has shown that moderate differences in temperature (–1.8 to –0.5˚C), with associated changes in salinity, correlate with differences in community composition for protists and bacteria [17]. Culture studies of polar microalgae have shown physiological adaptations to shifting environments, including changes in ribosome and protein abundance, enzyme activity, fatty acid content and composition, production of ice-binding proteins and exopolymers, and alterations in compatible solute concentrations (for review, see [23, 24]), but observations from mixed natural communities remain limited.

Compatible solutes, or osmolytes, are small organic molecules in a cell’s cytosol that maintain turgor pressure and stabilize enzymes; they also play other roles [25, 26]. For example, they act as cryoprotectants in polar microalgae by reducing the intracellular freezing point and maintaining protein hydration spheres [27]. Polar diatoms in culture accumulate compatible solutes to high intracellular concentrations (~1 M) in a taxon-specific manner and alter concentrations as a function of temperature or salinity [28–33]. Direct measurements of compatible solutes in polar environments are rare, but previous work suggests that many are similarly abundant in diatom-dominated sea ice and sea-ice diatom cultures [30].

Compatible solutes contribute to the larger pool of intracellular metabolites. Although marine particulate organic matter is composed largely of macromolecules [34], small polar metabolites, including compatible solutes, are the main component of the aqueous cytosol of cells in particulate organic matter [35]. These metabolites are considered a currency of the microbial loop [36], with roughly half of annual net primary production in the ocean rapidly cycling through as labile dissolved organic carbon compounds (DOC; e.g. metabolites) that are oxidized by heterotrophs [37]. Metabolites serve as carbon, nutrient, and energy sources for heterotrophic bacteria [38, 39], but also mediate phytoplankton-bacteria interactions, serve as predator repellants, manage redox stress, and more [35, 40, 41]. Measurements of the diversity and concentration of small polar metabolites in natural marine communities are limited [42–44], particularly for polar regions [30], and the plasticity of metabolite concentrations is largely unknown [45]. Intracellular metabolite pools can shift substantially in response to microbial interactions [38], making measurements on whole communities valuable alongside single-organism laboratory studies. Thus, in situ measurement of particulate metabolites and their sensitivity to temperature and salinity change will enhance our understanding of microbial physiology during seasonal transitions between polar marine habitats and the resultant impacts on community membership and chemical composition of marine organic matter.

Here, we characterize community structure and particulate metabolite profiles of bacterial and protist communities in sea-ice meltwater, seawater, and sea ice during austral spring along the WAP, recognizing that both the taxonomic composition of microbial communities and the chemical inventory of metabolites impact the flux of carbon and energy though polar food webs. We also examine the flexibility of metabolite pools in the surface seawater community under temperature and salinity conditions that mimic the formation and melt of sea ice. As polar oceans experience rapid warming, increased freshwater inputs, and shifts in seasonal ice formation and melt, this research serves to deepen our understanding of the potential responses of the microbial communities.

## Materials and methods

### Field sample collections

Sea-ice meltwater, seawater, and sea-ice samples were collected near Palmer Station, Anvers Island, Antarctica in November of 2018 (Table 1). Surface (< 1 m) seawater samples were collected into acid-clean carboys at 5 intervals over an 11-day period (November 8–19) in open water off Bonaparte Point (Station B of the LTER sampling grid; [46]). Sea-ice meltwater was collected into acid-clean carboys as surface water adjacent (< 1 m) to a melting pan of landfast sea ice located in Hero Inlet next to Palmer Station and the Marr Glacier. Sea-ice cores were opportunistically collected in this area from the R/V *Lawrence M. Gould* using a Kovacs MARK II ice auger during LMG1810 (Cruise 10.7284/907972). For all ice cores, the bottom 5-cm sections were melted in the dark at 4 °C into prefiltered (0.2 μm) seawater to avoid osmotic shock and cell lysis. Final salinities of ice samples following this melt procedure are provided in Table 1. Samples were collected in triplicate for all analyses unless noted otherwise (Table 1).

**Table 1:** Summary of samples collected and analyzed in this study.

Date	Sample type	Sample name	Latitude	Longitude	*n*	T (˚C)	S (ppt)	PAR (μmol m^−2^ s^−1^)
2018-11-05	Sea-ice meltwater	Meltwater	−64.78	−64.05	3	–0.7	25.0	320
2018-11-08	Seawater	SW_08	−64.78	−64.07	3	–0.9	35.7	305
2018-11-12	Seawater	SW_12	−64.78	−64.07	3	–0.7	35.2	375
2018-11-15	Seawater	SW_15	−64.78	−64.07	3	–0.3	35.2	1100
2018-11-17	Seawater	SW_17	−64.78	−64.07	3	–0.1	35.2	850
2018-11-19	Seawater	SW_19	−64.78	−64.07	3	0	35.2	700
2018-11-19	Sea ice	Sea ice _1	n.d.	n.d.	3	n.d.	12.0^a^	n.d.
2018-11-14	Sea ice	Sea ice _2	n.d.	n.d.	1	n.d.	15.0^a^	n.d.
2018-11-20	Sea ice	Sea ice _3	n.d.	n.d.	1	n.d.	20.0^a^	n.d.
2018-11-20	Incubation treatment Meltwater	Meltwater_T-S	n.a.	n.a.	3	3	21.0	100
2018-11-20	Incubation treatment Seawater	SW_T-S	n.a.	n.a.	3	0	35.0	100
2018-11-21	Incubation treatment Sea ice	Sea ice_T-S	n.a.	n.a.	3	–3	52.0	100

*n.d.* not determined, n.a., not applicable.

^a^Salinity measured following ice-core melt into filtered seawater.

### Temperature and salinity incubations

On the 12th of November 2018 (sample SW_12), we collected additional seawater for incubation experiments that simulated temperature(T)-salinity(S) conditions of sea-ice meltwater (3˚C and salinity 21, designated Meltwater_T-S), ambient seawater (0˚C and salinity 35, SW_T-S), and sea ice (–3˚C and salinity 52, Sea ice_T-S). Triplicate 10-L polycarbonate carboys were used for each treatment. All samples were enriched with f/2 nutrients with silica [47] to prevent potential nutrient limitation in faster-growing treatments of our closed-bottle experiments, where nutrients cannot be replenished by mixing as they are in situ. Maintaining nutrient replete status in all of our samples was necessary to discern the effects of temperature and salinity, rather than nutrient availability, on community and metabolome compositions. Carboys were incubated for approximately 10 days at 100 μmol photons m^–2^ s^–1^ light on a 20:4 h light:dark cycle. Incubations were subsampled daily for growth and harvested during exponential growth for the measurements detailed below. For details on incubation set up and monitoring, see Supplementary information.

### Sample processing

Field and incubation samples were processed for particulate metabolomics, DNA sequencing, chlorophyll *a* (Chl *a*), particulate organic carbon and nitrogen (POC, PN), DOC, particulate and dissolved extracellular polysaccharides (pEPS, dEPS), and major nutrients (NO_3_^-^, NO_2_^-^, NH_4_^+^, SiO_4_^4-^, PO_4_^3-^). All methods for the collection and processing of these samples are described in Supplementary information. Temperature, salinity, and photosynthetically active radiation (PAR) were measured using a digital thermometer, refractometer, and Walz US-SQS/L spherical quantum sensor with ULM-500 light meter, respectively.

### Metabolite extraction and LC-MS analysis

Metabolites within the particulate pool were extracted from samples filters using a modified Bligh and Dyer extraction, separated via liquid chromatography with a Waters Acquity I-Class UPLC equipped with either a reversed phase or hydrophilic interaction liquid chromatography column, ionized with electrospray ionization with a Thermo Q-Exactive mass spectrometer, and data was acquired in full scan mode as modified from Boysen et al. (2018). Further details on metabolite extraction and LC-MS analysis are provided in Supplementary information.

### Metabolomic data processing

Metabolite peaks obtained from mass spectrometry were integrated using Skyline for small molecules [48]. Full and abbreviated compound names are listed in Table S1, with abbreviated names used in figures throughout. Integrated peak areas were subject to quality control (Table S2) and normalized to the peak area of internal standards (Table S3) using best-matched internal standard normalization [49]. For details, see Supplementary information.

### Metabolite concentration calculations

Absolute concentrations of 134 compounds were calculated or estimated from peak areas using commercially available standards run in the same batch as our samples, similar to previous work [31, 42, 44]. Full quantification methods are provided in Supplementary information and summarized in Table S1.

### DNA extraction, sequencing, and processing

All methods for DNA extraction, high throughput sequencing, and analysis for unique amplicon sequence variants (ASVs) are given in Supplementary information. The Inverse Simpson alpha diversity index was calculated using the phyloseq package in R [50] following Callahan et al. [51].

### Statistical approaches

All methods for evaluating statistical differences in community structure, metabolite composition, metabolite concentrations, and ancillary measurements between samples are described in Supplementary information. For all statistical analyses, a probability level of ≤ 0.05 was used to determine statistical significance with *p* values corrected for false discovery rate (FDR) [52] where appropriate.

## Results

### Physical environment

Temperature and salinity in our meltwater, seawater, and sea-ice field samples were narrower in range than those imposed in the incubation treatments. Field temperatures for meltwater and seawater ranged between –0.9 and 0˚C, while salinity was approximately 25 and 35, respectively (Table 1). PAR varied substantially in the field, ranging from 305 to 1100 µmol photons m^−2^ s^−1^ (compared to 100 µmol photons m^−2^ s^−1^ in the treatments; Table 1). Though not measured directly in sea-ice samples, in situ temperature and brine salinity are presumed to be close to seawater [53] and PAR is presumed to be lower than surface seawater due to the attenuation of light in ice. The final salinity of sea-ice samples following the melt procedure for sampling was lower than seawater (Salinity 12–20; Table 1).

### Biological measurements

**Fig. 1:**
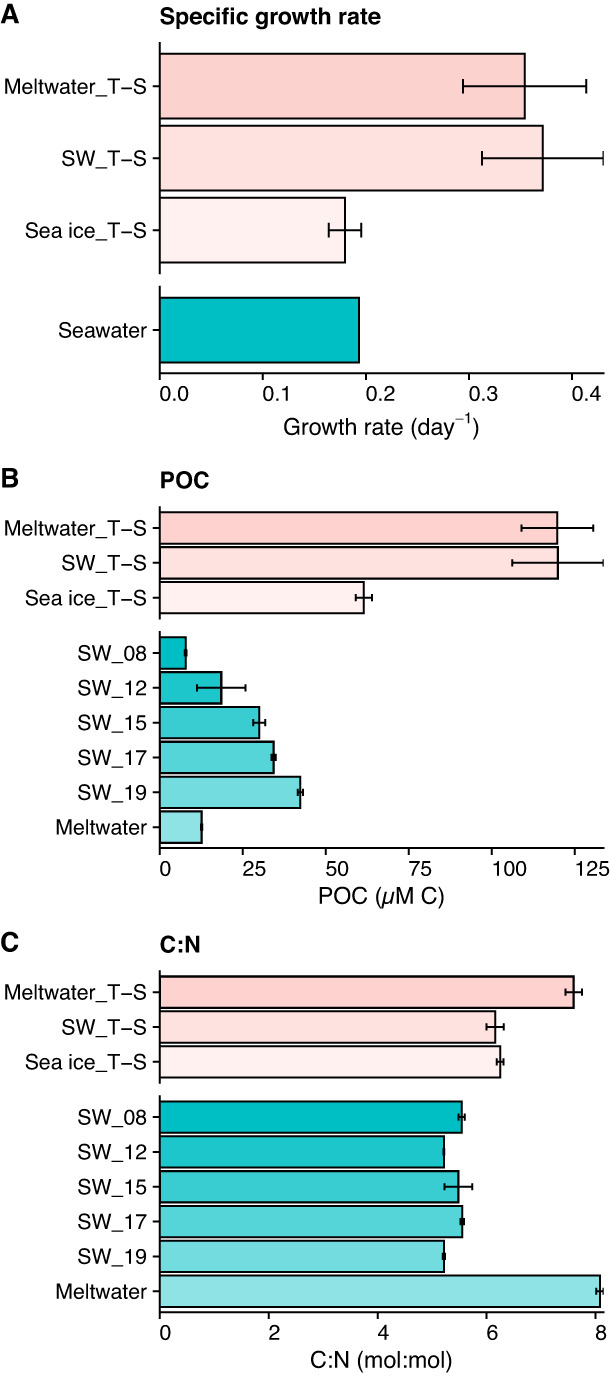
General parameters for incubation and field samples. **A** Specific growth rate (day^–1^) in incubated samples based on exponential change in Chl *a* fluorescence (during days 5–9 for Meltwater_T-S and Seawater_T-S, and days 6–10 for Sea ice_T-S) and in POC (days 6–10) for the seawater field samples; **B** concentration of particulate organic carbon (POC in μM C); and **C** molar ratio of C:N. Error bars represent standard deviation of the mean (*n* = 3). For all plots, y-axis break separates incubation treatment samples on the top (pink) and field samples on the bottom (aqua). Growth curves used to generate specific growth rate are provided in Fig. S1a and b; full data are available in Table S4. Note that we do not have POC or C:N measurements to pair with sea-ice field samples.

Surface seawater from Nov 12 was used to inoculate the incubations. After an initial lag, photosynthetic growth was detected in all incubation treatments with specific growth rates of approximately 0.3 d^−1^ for Meltwater_T-S and SW_T-S and 0.2 d^−1^ for Sea ice_T-S (Fig. 1A, S1a). The seawater community sampled over Nov 8–19 also showed an increase in biomass (Chl *a* and POC) with an estimated specific growth rate of approximately 0.2 d^−1^ (Fig. 1A, S1b, Table S4). A slight drawdown of nutrients over the sampling period was observed in both the seawater samples and the incubation treatments, though none reached limiting conditions and there was no clear trend based on treatment condition (Table S4). This maintenance of nutrient replete status across samples allowed for comparison of metabolome and community compositions between treatments with different growth rates based on the impact of temperature and salinity change. Meltwater had comparable biomass (POC, Fig. 1B) and nutrient concentrations (Table S4) to seawater. C:N ratios were generally low (5.2–5.6 in seawater and 6.2 in SW_T-S and Sea ice_T-S), with slightly higher values in meltwater and Meltwater_T-S (7.6 and 8, respectively; Fig. 1C).

### Alpha diversity

**Fig. 2:**
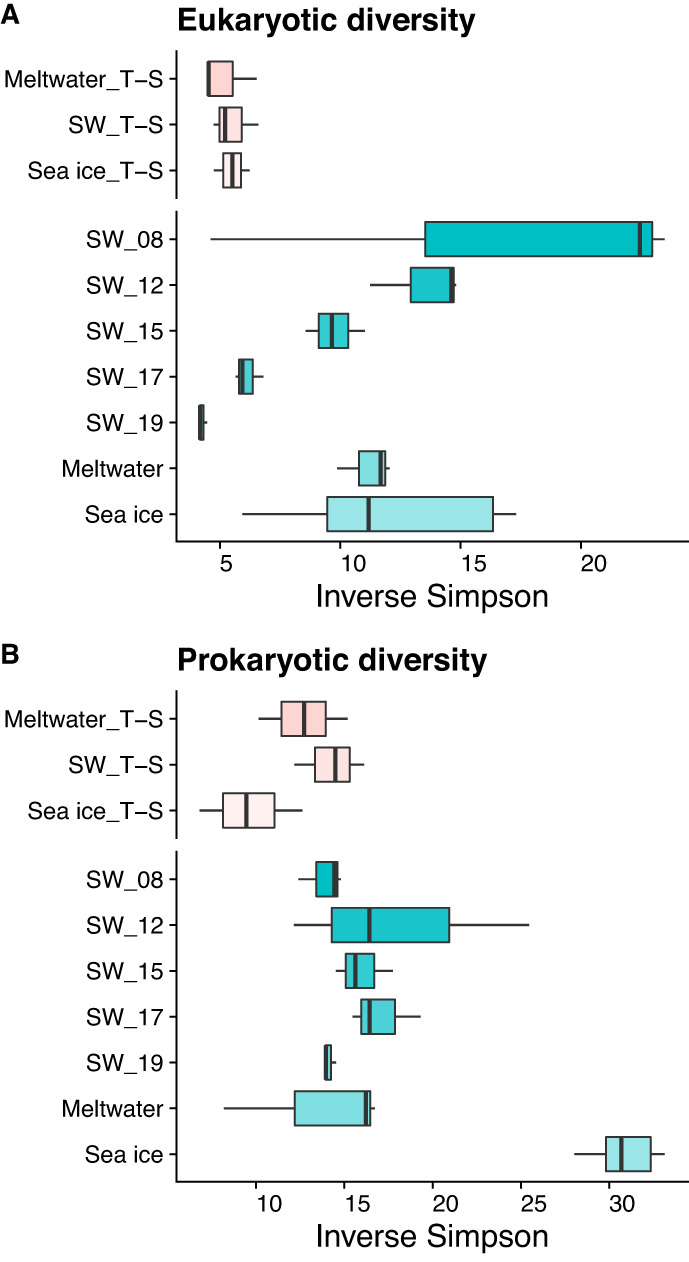
Alpha diversity in incubation and field samples. Inverse Simpson indices of alpha diversity for (**A**) the eukaryotic community and (**B**) the prokaryotic community in both incubation (pink) and field (aqua) samples. In the box plots, the total data range, median, and the 25–75% quartile range (box) are shown. For all plots, y-axis break separates incubation treatment samples on the top and field samples on the bottom.

Eukaryotic diversity in seawater between Nov 8–12 was initially high (Inverse Simpson’s index of 14 for the incubation inoculum SW_12) and decreased throughout the field sampling period and in incubation bottles as the incubation progressed (Fig. 2A), reaching similarly low diversity (approximately 5) within a similar timeframe (7 and 8–9 days, respectively). Sea ice and meltwater eukaryotic diversity fell within the range found in seawater. The prokaryotic community did not show a clear change in diversity over the sampling period in either seawater or the incubation treatments (Fig. 2B), and meltwater diversity was similar to that in seawater. The prokaryotic sea-ice community, however, was considerably more diverse (Inverse Simpson’s index of 30) than all other communities sampled (*p* « 0.001).

### Community composition

**Fig. 3:**
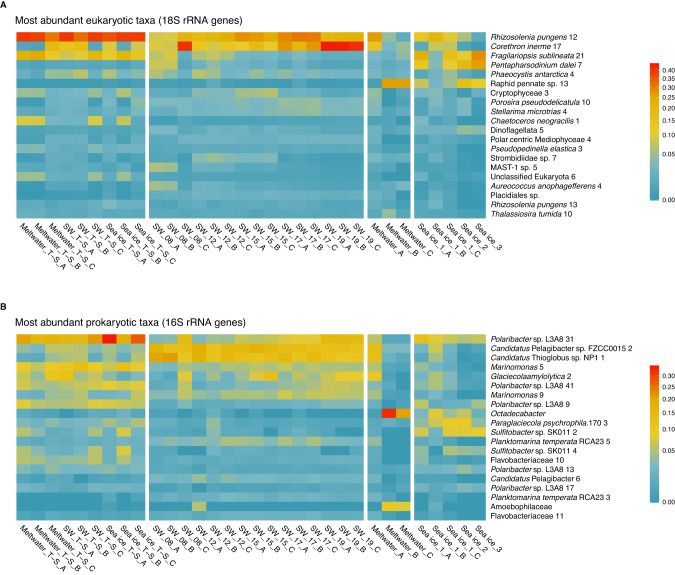
Community composition of incubation and field samples. Color-scaled relative abundance of the 20 most abundant (**A**) eukaryotic (18S) closest completed genomes (CCGs) and closest estimated genomes (CEGs) and (**B**) prokaryotic (16S) CCGs and CEGs. Vertical white spaces between samples separate incubation samples for all sample types (left) from field samples by sample type; sample designations _A, _B and _C indicate triplicate samples. Distinct ASVs assigned the same taxonomic name are differentiated by a number following the name (e.g. *Rhizosolenia pungens* 12 versus *Rhizosolenia pungens* 13). Note that the color bars have a square root transformation. Full data available in Tables S5 and S6.

Diatoms (Bacillariophyta) dominated the eukaryotic community numerically in all samples, regardless of habitat or treatment. The initial seawater community was approximately 50% diatoms, increasing to 80% over the sampling period for both seawater and incubation treatments (Fig. S2). The diatom *Rhizosolenia pungens* dominated in all incubations and increased in seawater (from SW_08 to SW_17), though the diatom *Corethron inerme* eventually dominated in seawater (SW_19; Fig. 3A). In contrast, the taxonomic composition of sea ice was mixed, with higher proportions of the diatom *Fragilariopsis sublineata* and the dinoflagellate *Pentapharsodinium dalei*, while most meltwater samples were dominated by a raphid pennate diatom. The prokaryotic community was largely bacterial, with the classes Gammaproteobacteria, Alphaproteobacteria, and Flavobacteriia making up the majority of ASVs (Fig. S3). The genus *Polaribacter* was abundant across seawater (increasing in abundance with time), sea-ice, and all incubation samples (Fig. 3B). *Polaribacter* ASVs were less abundant in the meltwater field samples, which were dominated by *Octadecabacter*. Seawater also had high contributions of *Candidatus* Pelagibacter and *Candidatus* Thioglobus (decreasing in abundance with time). Sea-ice bacterial communities were mixed, with large contributions from *Polaribacter*, *Sulfitobacter*, and *Paraglaciecola*. Archaeal ASVs were rare, making up 0.05–2% of total reads per sample, and dominated by the phylum Thaumarchaeota. Full ASV abundance data and taxonomic information for 18S and 16S rRNA gene sequencing, including archaeal ASVs, are available in Tables S5 and S6, respectively.

### Interdependency of prokaryotic and eukaryotic community structures

**Fig. 4:**
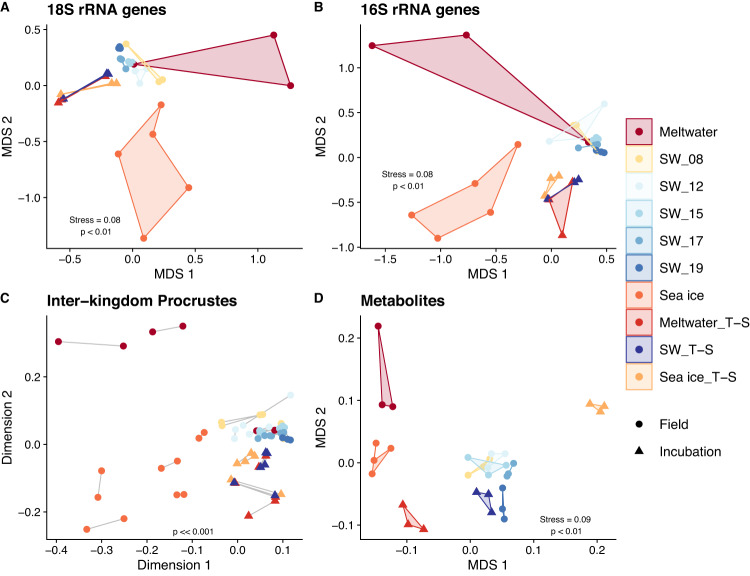
Multidimensional structure of community and metabolite composition in incubation and field samples. Non-metric dimensional scaling (NMDS) ordination, using Bray-Curtis dissimilarities, comparing (**A**) the eukaryotic (18S) composition and (**B**) the prokaryotic (16S) composition of each sample. **C** Procrustes analysis, where points represent individual samples, line connections between points represent eukaryotic and prokaryotic community composition from the same sample, and longer lines indicate greater within-sample dissimilarity between eukaryotic and prokaryotic community structure. **D** NMDS ordination, using Euclidean distance, comparing the metabolite composition of each sample. Metabolite concentrations (of 134 metabolites) are scaled to mole fraction of carbon. A version of this figure excluding metabolites that were added as part of f/2 nutrients (Cyanocobalamin and Vitamin B1) is available in Fig. S14). Colors indicate sample type. Full data for 18S, 16S, and metabolites are provided in Tables S5, S6, and S11, respectively.

NMDS ordinations of community structure paired with analysis of similarity (ANOSIM) tests displayed a clear separation of meltwater, seawater, and sea ice from one another (eukaryotic: R = 0.78, *p* « 0.001; prokaryotic: R = 0.79, *p* « 0.001), despite one meltwater replicate grouping with seawater (Fig. 4A, B). Seawater samples grouped together in ordination space, though community structure changed significantly over the course of seawater sampling (eukaryotic: R = 0.67, *p* « 0.001; prokaryotic: R = 0.40, *p* = 0.003). Incubation community structures during harvest were distinct from the initial seawater (eukaryotic: R = 0.61, *p* « 0.001; prokaryotic: R = 0.88, *p* « 0.001) but did not differ between treatments (eukaryotic: R = –0.08, *p* = 0.47; prokaryotic: R = 0.12, *p* = 0.23). Full ANOSIM results are listed in Tables S7 and S8.

NMDS ordinations revealed similar patterns within the eukaryotic and prokaryotic community structures (Fig. 4A, B). Symmetric Procrustes analysis revealed a strong, significant congruency among these communities (*m*^2^ = 0.14, *p* « 0.001), whereby samples with similar eukaryotic community structures were likely to share similar prokaryotic community structures (Fig. 4C). Correlation analyses based on the Spearman correlation of centered log-ratio-transformed data indicated numerous inter-domain co-occurrences. Across the top 20 eukaryotic and prokaryotic ASVs in our sample set (32 samples), 350 ASV pairings correlated significantly, with 189 correlating positively and 161 negatively (Fig. S4a, Table S9). These pairings included positive correlations between abundant diatom ASVs *Rhizosolenia pungens*.12 (ρ = 0.76, *p* = 3.49 ×10^–7^) and *Chaetoceros neogracilis*.1 (ρ = 0.75, *p* = 9.98 ×10^–7^) with *Polaribacter* sp. L3A8.31. Across our field samples (23 samples), significant positive correlations between ASVs aligned largely by habitat type (Fig. S4b): taxa abundant in sea-ice and meltwater samples (e.g. *Sulfitobacter*, *Octadecabaceter*, *Fragilariopsis sublineata*) correlated with each other, while taxa abundant in seawater samples (e.g. *Candidatus* Pelagibacter, *Candidatus* Thioglobus, *R. pungens*) correlated with each other in a separate cluster. Full correlation results can be found in Tables S9 and S10.

### Metabolite pools in polar environments

Particulate pools of 134 known metabolites were quantified (see Table S11). NMDS ordinations paired with ANOSIM tests showed that metabolite profiles clearly separated the meltwater, seawater, and sea-ice samples from each other (R = 0.99, *p* « 0.001), including a weaker but significant shift over the seawater sampling period (R = 0.61, *p* = 1×10^–^^4^; Fig. 4D). This distinction by habitat followed the observed pattern in community structure, with samples similar in metabolite composition tending to be similar in eukaryotic and prokaryotic community structure as well (Fig. S5). Unlike community structure, metabolite profiles for incubated samples separated strongly according to treatment (R = 0.97, *p* = 0.003), with the control treatment (SW_T-S) grouping closely with the in situ seawater samples. When grouped by salinity status, the samples ordered across MDS1, with fresher field samples (meltwater and sea ice) grouping significantly with the Meltwater_T-S samples as compared to the ambient-salinity seawater or high-salinity Sea ice_T-S samples (Fig. S6; R = 0.89, p « 0.001). Full ANOSIM results are provided in Table S12.

**Fig. 5:**
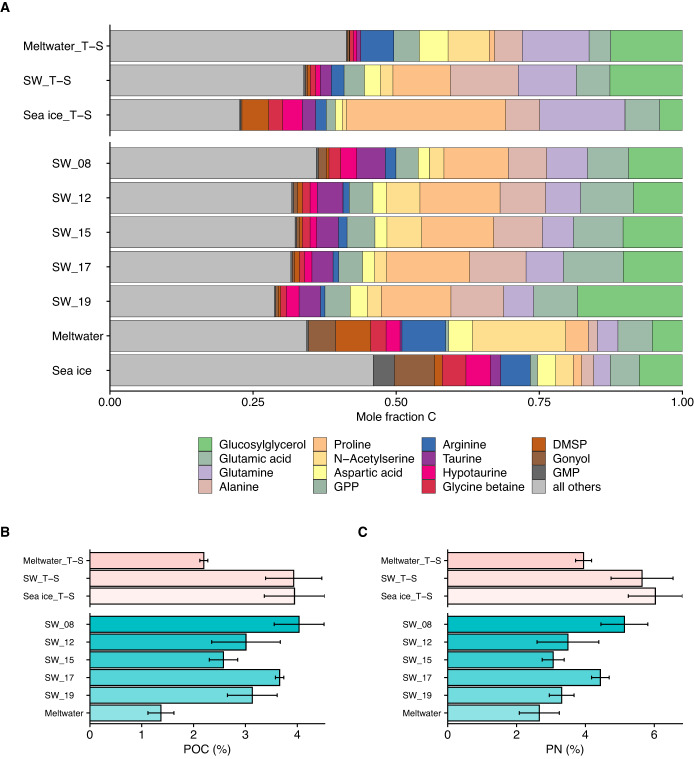
Metabolite composition of particulate matter in incubation and field samples. **A** Metabolite abundance presented as mole fraction of carbon of total identified metabolites [134] across the incubation and field samples. Average of triplicates are shown. The most abundant 15 molecules for each sample are color-coded, with “all others” (gray) containing the sum of the remaining quantified metabolites [119]. Total quantified metabolite concentration as the percentage of (**B**) particulate organic carbon (POC) and (**C**) particulate nitrogen (PN), where error bars represent standard deviation of the mean (*n* = 3). For all plots, y-axis break separates incubation treatment samples on the top and field samples on the bottom. For (**B**) and (**C**), color denotes incubation (pink) versus field (aqua) samples. Full data available in Table S11; individual metabolite contributions as %POC and %PN, available in Figs. S8 and S9, respectively. Note that we do not have POC or PN to pair with sea-ice field samples.

Total quantified particulate metabolite concentration (as molar carbon concentration, nM C) ranged broadly between field sample types, from approximately 170 nM C in meltwater to 315–1325 nM C in seawater (Fig. S7; Table S14). In our seawater samples, this equates to 2.6–4.0% of POC and 3.1–5.1% of PN, with no clear pattern over the sampling period (Fig. 5B and C, S8 and S9). While SW_T-S and Sea ice_T-S metabolite contributions to POC and PN were similar to seawater (approximately 4% POC and 6% PN), both the incubation and field meltwater metabolite pools made up a smaller %POC (approximately 2 and 1.25%, respectively) and %PN (approximately 4 and 2.25%, respectively) (Fig. 5B, C). The 20 most abundant metabolites accounted for about 70% of the total quantified metabolite pool in all samples (Fig. 5A), with compatible solutes (e.g. glucosylglycerol, proline, glycine betaine [GBT], dimethylsulfoniopropionate [DMSP].) and free amino acids (e.g. glutamic acid, glutamine, alanine) among the most abundant, similar to previous work [31, 42, 44]. There were clear differences in particulate metabolite composition between the environments sampled and between incubation treatments (Fig. 5A). For example, seawater samples had high contributions (on a mole fraction carbon basis) from glucosylglycerol, proline, glutamic acid, glutamine, and alanine, while sea-ice and meltwater samples had high contributions from arginine and from gonyol and N-acetyl serine, respectively (Figs. 5A and S10). In total, 95 of 134 metabolites contributed significantly to the separation of samples in NMDS ordination space (statistics available in Table S15).

### Metabolite temperature and salinity sensitivities

**Fig. 6:**
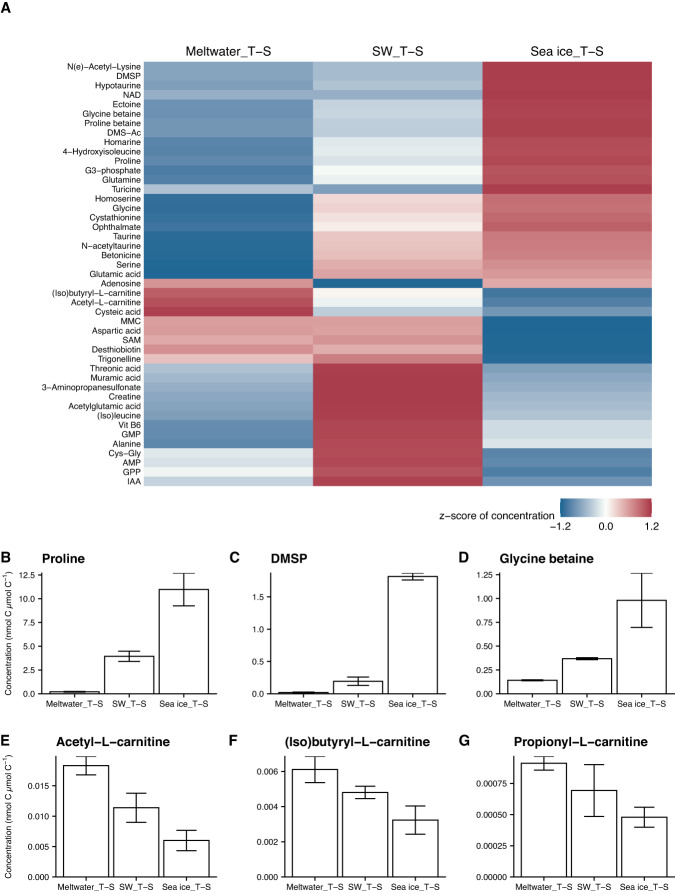
Particulate metabolite responses to temperature and salinity change during the incubation experiments. **A** Heat map color-coded by z-score standardized concentrations of 134 metabolites (nmol metabolite C µmol C^–1^), arranged by average linkage hierarchical clustering of Euclidean distance (dendrogram of clustering available in Fig. S11), for the three different treatments. Compounds listed were each significantly different (*p* < 0.05) with treatment, as determined by false discovery rate-corrected *p* values from one-way ANOVAs (detailed in Table S16); compounds not significantly different (*p* > 0.05) are available in Fig. S12. Concentration (nmol metabolite C µmol C^–1^) of compatible solutes in the incubations, grouped by treatment, for (**B**) proline, (**C**) DMSP, and (**D**) glycine betaine, and of acylcarnitines for (**E**) acetyl-L-carnitine, (**F**) Isobutyryl-L-carnitine, and (**G**) Propionyl-L-carnitine. Error bars represent standard deviation of the mean (*n* = 3).

Normalizing to POC (nmol metabolite C µmol C^–1^), the concentration of 45 (34%) metabolites differed significantly across temperature and salinity treatments (Fig. 6A; Table S16). Hierarchical clustering (Figs. 6A and S11) indicated that half of those metabolites [23] increased under cold and salty conditions (Sea ice_T-S) and decreased under warmer and fresher conditions (Meltwater_T-S) compared to seawater controls (SW_T-S). Many of these metabolites are known osmolytes (proline, DMSP, GBT). Proline was the most abundant metabolite quantified in our experiment (up to 1.3% of the total POC pool or 29% of the total metabolite C pool in Sea ice_T-S) and responded strongly to temperature and salinity change: proline was approximately 3 times higher in Sea ice_T-S (*p* = 0.00042), and 20 times lower in Meltwater_T-S (*p* = 0.011), compared to SW_T-S (Fig. 6B). DMSP and GBT were less concentrated than proline (up to 0.3% and 0.1% of POC, respectively) but responded similarly to temperature and salinity change (Fig. 6C, D).

Many other compatible solutes were present at much lower concentrations (0.00005% to 0.1% of POC) but clustered with proline, DMSP, and GBT, and responded similarly to temperature and salinity change. These include homarine, hypotaurine, taurine, homoserine, dimethylsulfonioacetate (DMS-Ac), ectoine, proline betaine, and betonicine (Fig. 6A; Table S11). A few compatible solutes (isethionic acid, gonyol, DHPS) did not show a significant response to temperature and salinity (Fig. S12). Only three metabolites were uniquely enriched under Meltwater_T-S conditions: cysteic acid, acetyl-L-carnitine, and (iso)butyryl-L-carnitine (Fig. 6A, E, F). Propionyl-L-carnitine followed the same pattern, though the response was not statistically significant (Figs. 6G and S12).

## Discussion

In this study, the microbial communities of three polar habitats (meltwater, seawater, and sea ice) with distinct physicochemical properties showed unique community structures and taxonomic compositions (Figs. 2–4 and S2–S3). This finding agrees with previous observations [14, 17], with warming spring sea ice associated with mixed pennate diatoms (here, largely *Fragilariopsis*) and flagellates (here, mainly *Pentapharsodinium*). Centric diatom taxa (*Rhizosolenia* and *Corethron*) became dominant in seawater and all incubation treatments, as commonly observed during phytoplankton blooms in WAP seawater [54–56]. Community structure differed slightly between in situ seawater and our incubations (Fig. 4A, B), which could be driven by the artificial environment of our incubations which included LED lighting and the addition of f/2 nutrients. However, both had similar dominant algal and bacterial taxa (*Rhizosolenia* and *Polaribacter*, respectively; Fig. 3), suggesting that our incubations remained representative of the seawater community. Numerically, copiotrophic bacteria (*Polaribacter*) overtook oligotrophic taxa (*Candidatus* Pelagibacter [SAR11] and *Candidatus* Thioglobus [SUP05]) during the onset of the phytoplankton bloom in situ (Fig. 3B), as shown previously along the WAP [57] and in the open Southern Ocean [58]. This trend, likely due to faster growth rates of copiotrophs with high availability of labile dissolved organic matter (DOM) [59], was amplified in our closed bottle incubations where DOC was approximately 15 times more concentrated than seawater levels (Table S4 and Fig. S1e).

Microbial community composition is often sensitive to temperature and salinity in sea ice [17] and seawater [60]. Temperature and salinity did not drive community restructuring during our 10-day incubations (Figs. 2–4), possibly due to the length of the experiment or the stability of control variables (light, nutrients, habitable pore space) that often vary alongside temperature and salinity in the environment. Previous studies on polar seawater also suggest that the influence of temperature and salinity on community structure is highly dependent on the starting community composition [60–62]. Thus, changes in the timing of sea-ice formation and melt could lead to differences in the starting seawater community that will experience temperature and salinity shifts and may alter the resultant microbial community composition.

Congruency between eukaryotic and prokaryotic community structure across all of our samples (Fig. 4C) indicates a consistent coupling between these domains. Algal production and bacterial heterotrophy are tightly coupled in other marine environments [63–65], and specific eukaryotic and prokaryotic taxa have been observed to co-occur in Antarctic sea ice [17, 66] and seawater [67]. Many positive correlations between eukaryotic and prokaryotic ASVs identified here (Fig. S4) are similar to those observed in an Arctic culture study [68] (e.g. a *Chaetoceros* diatom with the flavobacteria *Polaribacter* and a *Fragilariopsis* diatom with the alteromonad *Paraglaciecola)*. The specificity of co-occurrences (e.g., *Fragilariopsis sublineata* did not correlate positively with another alteromonad genus, *Glaciecola*) could reflect taxa-specific use of algal exudates by heterotrophic bacteria [68–73]; it could also reflect shared environmental optima rather than direct metabolite exchanges or symbioses. Detecting these inter-domain co-occurrences provides a starting point to target algal-bacterial pairs for future studies of microbial interdependencies in polar oceans.

Particulate community metabolomes were distinct across WAP meltwater, seawater, and sea ice (Figs. 4D and 5), following the pattern of community structure (Fig. S5). These metabolome distinctions were likely driven by a combination of habitat-unique taxonomic composition and physicochemical conditions (e.g., salinity, temperature, light). This explanation is consistent with previous seawater and culture studies, where metabolomes reflected community composition or individual taxa identity [31, 44, 74] but were also shaped by physiological responses to environmental conditions [30, 75]. For example, the dinoflagellate compatible solute gonyol [44, 76] was proportionally enriched in our dinoflagellate-rich sea-ice and meltwater samples (Figs. 5 and S10) despite habitat differences in physicochemical factors (Table 1). In contrast, despite only minor changes in microbial composition throughout our incubation, community metabolomes differed significantly between treatments (Figs. 4D, 5, and 6), suggesting that the seawater taxa present had the metabolic flexibility to adapt to new temperatures and salinities and maintain growth. The experimental control samples maintained at ambient seawater temperature and salinity conditions had markedly similar metabolite profiles and concentrations to the in situ seawater samples (Figs. 4D and 5), which suggests we sampled a nutrient replete system in the early stages of a phytoplankton bloom, where the addition of nutrients alone did not significantly impact metabolome composition within the experiment. Since metabolome composition and concentrations did vary between treatments with the same nutrient availability, we instead conclude that the observed differences were driven largely by temperature and salinity. In this context, our metabolome results suggest that changes in temperature and salinity could significantly alter polar ocean carbon cycling and microbial interactions mediated by metabolites, even in taxonomically stable and nutrient replete communities. The summed concentrations of metabolites in WAP surface seawater measured here (315–1325 nM C) were higher than those measured in surface marine particles across a North Pacific Ocean transect (68–234 nM C; [44]), but the metabolite contributions to total POC and PN were largely similar, suggesting that the differences could be attributable to the higher biomass in our samples.

Metabolite profiles from the seawater and meltwater incubation treatments were largely similar to their in situ counterparts (Fig. 4D). The Sea ice_T-S incubation did not group with the sea-ice samples, likely because the incubation conditions resemble wintertime sea ice (higher salinity and lower temperature than seawater), while the brine salinity and temperature of warming spring sea ice we sampled were likely closer to seawater or approaching meltwater upon melt for sampling. This colder and saltier treatment (Sea ice_T-S) was strongly distinguished by an increased contribution of proline (Fig. S10), a known compatible solute in polar microbe cultures [21, 28–30]. Proline was the most abundant metabolite quantified in our sample set, with seawater concentrations (0.5–1.1 nmol C µmol C^–1^) similar to those measured in Arctic sea ice (0.5–1.5 nmol C µmol C^–1^; [30]). Proline concentration in POC was elevated 3-fold in the Sea ice_T-S (Fig. 6), a similar magnitude as in culture studies of two polar diatoms: *Fragilariopsis cylindrus* [28] and *Nitzschia lecointei* [30]. Determining the exact source of this proline (or any metabolite) within POC, which includes total community biomass and detritus, is not possible given the methods of this study, but an increase in metabolite concentration in particulate matter reflects that sources of the metabolite (e.g. biosynthesis, uptake from DOM) are greater than sinks (e.g. catabolism, release as DOM).

Many other metabolites also increased in concentration under cold and salty conditions and decreased in warmer and fresher conditions (Fig. 6), supporting their potential roles in cryo- and osmoprotection [25, 77]. Some of these compounds are likely eukaryotic osmolytes (DMSP; [78–80]), but others are often associated with bacterial taxa (ectoine; [81–83]), reflecting a widespread community response to temperature and salinity change. Along with proline, three of these compounds are abundant in polar diatom cultures and show a similar response to cold and saline conditions: DMSP [30, 32], GBT [30, 33], and homarine [29, 31]. However, the majority have never been quantified or confirmed to be temperature- and salinity-sensitive in culture or in mixed polar marine microbial communities. Thus, the present study has uncovered numerous compounds that deserve more focus as potential compatible solutes (e.g. homoserine, ectoine, DMS-Ac, N(e)-acetyl-lysine; Fig. 6A) in polar contexts, where existing seasonal and future climate-altered shifts in temperature and salinity may impact the standing stocks and cycling of these labile compounds.

Seawater freshening left detectable imprints on organic matter across our samples, with the metabolomes of melt-influenced communities (Meltwater_T-S, meltwater, sea ice) more similar to each other than to higher salinity samples (seawater, SW_T-S, Sea ice_T-S; Fig. S6). Three acylcarnitines—acetyl-L-carnitine, (iso)butyryl-L-carnitine, and propionylcarnitine—accumulated under warm and fresh conditions in our incubation (Fig. 6E–G) and contributed to the separation of field meltwater samples in multivariate space (Fig. S10). Acylcarnitines participate in the transport of fatty acids across mitochondrial membranes for β-oxidation [84], and their accumulation may indicate changes in storage lipid pools, such as the degradation of fatty acids [85]. This possibility aligns with our observation of decreased free concentrations of three essential polyunsaturated fatty acids (PUFAs; arachidonic acid, eicosapentaenoic acid, and docosahexaenoic acid) in low-salinity samples (Fig. S13) and previous observations of PUFA regulation by low salinity in diatoms [86, 87] and Antarctic phytoplankton [88]. Seawater freshening could thus have important consequences for Antarctic food webs, as the PUFAs produced widely by autotrophs are essential in animal diets [89]. Fatty acid reorganization is also important for maintenance of membrane fluidity in polar microalgae under varying temperature [90] and potentially salinity [91], so this result may also be indicative of membrane restructuring in response to freshening and warming. Alternatively, acylcarnitines may play a role in diatom osmoadaptation or form as a secondary response to changes in amino acid synthesis [92, 93], but these possibilities have yet to be explored in polar microbes.

Freshening also drove reduced total metabolite concentrations, relative to POC and PN, and elevated C:N ratios (Figs. 1 and 5). This low-salinity signature was observed in both our incubation Meltwater_T-S (salinity 21) and field meltwater (salinity 25) despite differences in community composition, temperature, and nutrient concentrations (Figs. 3, 4A, and 4B; Table S4). Nitrogen limitation or carbon-rich EPS production does not appear to contribute to the higher C:N ratios at lower salinities (Table S4 and Fig. S1c, respectively). This pattern could be due in part to the release of abundant N-rich compatible solutes under hypoosmotic conditions, as shown for GBT in sea-ice algae cultures [33]. However, we estimate that the difference in metabolite concentrations between our freshened and ambient salinity treatments could only drive an approximate 1% change in C:N (6.16 versus 6.21 C:N; calculation in Table S17), much smaller than the difference measured here (23%; Fig. 1C). Differences in C:N were likely driven instead by shifts in abundant macromolecular pools (lipids, carbohydrates, protein) that make up approximately 80% of POC in surface seawater [34].

Although our data suggest that fluctuations in particulate metabolite concentrations are unlikely to alter bulk stoichiometry, they may rapidly alter the composition of labile DOM during increasingly common freshening events. Many metabolites can be respired by the organisms that produce them [42], but others can enter the surrounding dissolved pool via lysis, passive exudation, or active excretion, which has been observed on subsecond timescales in a polar bacterium during freshening [94]. Released N-rich compatible solutes, for example, can be taken up by other microbes and maintained for osmoregulation [33, 95] or catabolized [26, 39]. Microbial metabolism of compatible solutes can lead to the production of marine aerosols, such as monomethylamine or dimethylamine production from GBT via trimethylamine and trimethylamine-N-oxide [96, 97]. Additionally, sulfur-containing compatible solutes (gonyol, DMS-Ac) can inhibit the production of dimethyl sulfide, the primary natural sulfate aerosol precursor, from DMSP [98], suggesting that their release during freshening could alter dimethyl sulfide emissions in polar regions and thus climate regulation [99]. Specific molecular properties of marine DOM (e.g. molecular weight, nitrogen content) likely influence the composition and activity of the microbial community [65, 100], such that environmentally driven changes in DOM composition may alter organic matter cycling, organism interactions, and climate-active marine aerosol production along the WAP.

## Conclusion

In the present study, we show that the different marine habitats of sea ice, meltwater, and seawater harbor unique microbial communities distinct in both taxonomy and metabolite composition. Temperature and salinity change drove strong metabolome changes in these coastal WAP communities even in the absence of strong taxonomic change. Mechanistically, they exhibited a marked metabolic flexibility in response to rapid environmental change, the accumulation of compatible solutes under more wintry conditions, and the reverse depletion of those products with regulation of fatty acids under melt conditions. As model predictions in the WAP suggest earlier ice breakup and longer open water periods with warmer and fresher conditions, our results highlight that a subsequent restructuring of the microbial chemical inventory is possible, likely affecting the fate of organic matter and thus the balance between remineralization and sequestration of atmospheric CO_2_.

### Supplementary information

Supplemental Methods

Supplemental Figures

Supplemental Tables

## Data Availability

Supporting datasets are provided in the Supplementary Information (Datasets S1–S19). Sequence data are deposited in the National Centre for Biotechnology Information (NCBI) Sequence Read Archive (SRA). 18S and 16S rRNA gene amplicon data are deposited under the NCBI BioProject PRJNA942251. Metabolite data are deposited on Metabolomics Workbench (https://www.metabolomicsworkbench.org/data/index.php) under study ID ST002539. All data processing and analysis code is publicly available at https://github.com/hmdawson/PalmerStation2018_Targeted-master.
