# Supplementary material for: Microbial metabolomic responses to changes in temperature and salinity along the western Antarctic Peninsula
Source: ISME J. 2023 Sep 15;17(11):2035–46. doi: 10.1038/s41396-023-01475-0 (PMC10579395; doi:10.1038/s41396-023-01475-0)
Supplement: Supplementary file 1 — Supplemental Methods [file 41396_2023_1475_MOESM1_ESM.docx]

Microbial metabolomic responses to changes in temperature and salinity along the western Antarctic Peninsula.

Dawson, H.M.^1^, Connors, E.^2^, Erazo, N.^2,3^, Sacks, J. S. ^1^, Mierzejewski, V.^4^, Rundell, S.^1^, Carlson, L. T.^1^, Deming, J.W.^1^, Ingalls, A. E.^1^, Bowman, J.^2,3,5^, Young, J.N.^1,6^

1. School of Oceanography, University of Washington, Seattle, WA 98195, USA
2. ﻿Scripps Institution of Oceanography, UC San Diego, La Jolla, CA 92037, USA
3. Center for Marine Biodiversity and Conservation, UC San Diego, La Jolla, CA 92037, USA
4. School of Earth and Space Exploration, Arizona State University, Tempe, AZ 85287, USA
5. Center for Microbiome Innovation, UC San Diego, La Jolla, CA 92037, USA
6. Corresponding author

**Supplemental methods**

*Temperature and salinity incubation experiment set up and monitoring*

On 12 November 2018, we collected additional seawater (sample SW_12; Table 1) for incubation experiments that simulated temperature(T)-salinity(S) conditions of sea-ice melt (3˚C and 21 ppt, designated Meltwater_T-S), ambient seawater (0˚C and 35 ppt, SW_T-S), and sea ice (–3˚C and 52 ppt, Sea ice_T-S). Triplicate 10-L polycarbonate carboys were used for each treatment. All samples were enriched with f/2 nutrients with silica (1) and incubated for approximately 10 days at 100 μmol photons m^–2^ s^–1^ light on a 20:4 h light:dark cycle. All the carboys remained unfrozen throughout the experiment. Salinity was adjusted by dilution with MilliQ water or addition of artificial sea salts (top 6 salts by molar concentration from Enriched Seawater, Artificial Water (2)). All treatments contained the same volume of seawater inoculum (4.5 L) and reached the same total volume (9 L). Temperature was controlled using custom-built aquaria and monitored using Onset HOBO pendant data loggers. Incubations were subsampled daily for growth and harvested during exponential growth on day 8 for Meltwater_T-S and SW_T-S and day 9 for Sea ice_T-S for metabolomics, DNA sequencing, chlorophyll *a* (Chl *a*), particulate organic carbon and nitrogen (POC, PN), dissolved organic carbon (DOC), particulate and dissolved extracellular polysaccharides (pEPS, dEPS), and major nutrients (NO_3_^-^, NO_2_^-^, NH_4_, SiO_4_, PO_4_^3-^). Growth of photosynthetic organisms was monitored by daily changes in relative fluorescence units (RFU) using a Turner fluorometer.

*Sample filtration and processing*

Samples for particulate metabolomics were collected as described in Boysen et al. (2018). Briefly, samples were collected into polycarbonate containers, filtered onto 47 mm 0.2 µm ﻿polytetrafluoroethylene Omnipore Membrane filters using peristaltic pumping, stored in combusted aluminum foil, flash-frozen in liquid N_2_, and kept at –80°C until extraction. Methodological blanks were collected by passing filtrate over a second ﻿0.2 µm polytetrafluoroethylene filter to account for any dissolved or salt matrix contaminants, as in Heal et al. (2020). Samples for DNA sequencing were filtered onto 47 mm 0.2 µm Pall Supor polyethersulfone membranes and were then stored in their original sterile packaging, and frozen at –80°C until extraction.

Samples for POC, PN, DOC, Chl *a*, and major nutrients were collected and processed according to protocols of Marine Chemistry Laboratory at the University of Washington. Samples for POC and PN were filtered through combusted (450°C, 4 h) 25 mm glass fiber filters (GF/F, pore size 0.7 μm pre-combustion) and frozen at –80°C until analysis. Following fuming with HCl to remove inorganic carbon, CHN was measured on a CEC440 Elemental Analyzer (Leeman Labs) ﻿in the Marine Chemistry Laboratory (MCL) at the University of Washington. DOC was measured on 0.2 μm filtrate on a ﻿TOC-VCSH DOC analyzer (Shimadzu) in the MCL. pEPS and dEPS were measured in glucose-equivalents using the phenol-sulfuric acid method and converted to carbon-equivalents following Krembs et al. (2011). Samples for EPS were filtered through 0.4 μm polycarbonate filters; the filter and filtrate (< 0.4 μm) were used for pEPS and dEPS, respectively. Nutrients were analyzed in the MCL using a Technicon AutoAnalyzer II (6). Samples for Chl *a* were collected onto 25 mm glass fiber filters (GF/F, pore size 0.7 μm pre-combustion) and measured according to Welschmeyer (1994). Temperature, salinity, and photosynthetically active radiation (PAR) were measured using a digital thermometer, refractometer, and Walz US-SQS spherical quantum sensor -ULM-500 light meter, respectively.

*Metabolite sample extractions and analysis*

Metabolites were extracted from sample filters as detailed in Boysen et al. (2018). Briefly, metabolites were extracted using a modified Bligh-Dyer extraction in 1:1:2 methanol:water:dichloromethane, resulting in a polar aqueous (methanol and water soluble) and a non-polar organic (dichloromethane soluble) extract. Metabolites in the polar aqueous fraction were analyzed in this study, in addition to three fatty acids in the organic extract, as detailed below. A suite of internal standards (Table S3) were added before and after extraction as in Boysen et al. (2018) to use in downstream data normalization. Sample extracts were dried under clean N2 and reconstituted in 400 µL of Optima LC/MS grade water. Note that for the sample Sea ice_3 metabolite data is not shown throughout.

Metabolites from the polar aqueous extract were separated via liquid chromatography as in Boysen et al. (2018) with a Waters Acquity I-Class UPLC equipped with either a reversed phase (RP) or hydrophilic interaction liquid chromatography (HILIC) column, as detailed below. Both liquid chromatography configurations were coupled to a Thermo Q-Exactive (QE) mass spectrometer and data was acquired in full scan mode for compound quantification as modified from Boysen et al. (2018), as detailed below. Metabolites from the non-polar organic extract were similarly separated via liquid chromatography (Waters Acquity UPLC CSH C18 column) and analyzed on a coupled Thermo Q-Exactive (QE) mass spectrometer, as detailed below (referred to as “lipid analysis”).

*HILIC analysis liquid chromatography method*

For HILIC, a SeQuant ZIC-pHILIC column (5 um particle size, 2.1 mm x 150 mm, from Millipore) was used with 10mM ammonium carbonate in 85:15 water to acetonitrile (Solvent A) and 10mM ammonium carbonate in 85:15 acetonitrile to water (Solvent B) at a flow rate of 0.15 mL/min. The column was held at 100% B for 2 minutes, ramped to 64% A over 18 minutes, ramped up to 100% A over 1 minute, held at 100% A for 7 minutes, and equilibrated at 100% B for 22 minutes (total time is 50 minutes). The column was maintained at 30˚C.

*Reversed-Phase analysis liquid chromatography method*

For reversed-phase, a Waters Acquity UPLC HSS Cyano column (1.8 um particle size, 2.1 mm x 100 mm) equipped with a Acquity UPLC HSS Cyano guard column (1.8 um particle size, 2.1 mm x 5 mm) was used with 0.1% formic acid in water (Solvent A) and 0.1% formic acid in acetonitrile (Solvent B) at a flow rate of 0.4 mL/min. The column was held at 5% B for 2 minutes, ramped to 100% B over 18 minutes, held at 100% B for 2 minutes, and equilibrated at 5% B for 5 minutes (total run time is 25 minutes). The column was maintained at 35˚C.

Lipid analysis liquid chromatography method

For lipid analysis, a Waters Acquity UPLC CSH C18 column (1.7 um particle size, 2.1 mm x 150 mm) was used with 10mM ammonium formate in 60:40 acetonitrile to water and 0.1% formic acid (Solvent A) and 10mM ammonium formate in 90:10 isopropyl alcohol to acetonitrile and 0.1% formic acid (Solvent B) at a flow rate of 0.45 mL/min. Initial conditions were 90% A and 10% B. The column was ramped to 80% B over 33 minutes, ramped up to 90% B over 12 minutes, held at 90% B for 1 minute, and equilibrated to 90%A and 10% B for 6 minutes (total time is 52 minutes). The column was maintained at 65°C.

*QE (Orbitrap) mass spectrometry method*

Metabolites were measured using both hydrophilic interaction liquid chromatography (HILIC) and reverse phase chromatography (RP) on a Waters Acquity UPLC system coupled to a Thermo Qexactive HF (QE-HF) high-resolution mass spectrometer equipped with heated electrospray ionization (H-ESI).

The mass spectrometer was calibrated weekly in positive and negative mode using solutions provided by the manufacturer. For HILIC, a full scan method employing positive and negative switching was used with a scan range of 60 to 900 m/z and a resolution of 60,000. The capillary temperature was 320˚C, the H-ESI spray voltage was 3.5 kV, and the auxiliary gas heater temperature was 90˚C. The S-lens RF level was 65. Sheath gas, auxiliary gas, and sweep gas flow rates were maintained at 16, 3, and 1, respectively. For the quality control pooled samples, high-resolution MS scans were collected and separate injections were done for positive and negative ion modes.

For reverse phase, a full scan method was used with a scan range of 90 to 900 m/z and a resolution of 120,000 in positive mode. The capillary temperature was 320˚C, the H-ESI spray voltage was 3.8 kV, and the auxiliary gas heater temperature was 90˚C. The S-lens RF level was 65. Sheath gas, auxiliary gas, and sweep gas flow rates were maintained at 40, 10, and 1, respectively. For the quality control pooled samples, high-resolution MS scans were collected.

For lipid analysis, a full scan method was used with a scan range of 150 to 2000 m/z and a resolution of 120,000 in positive mode and negative mode (separate injections). The capillary temperature was 320°C, the H-ESI spray voltage was 3.8 kV, and the auxiliary gas heater temperature was 150°C. The S-lens RF level was 65. Sheath gas, auxiliary gas, and sweep gas flow rates were maintained at 40, 10, and 1, respectively. For the quality control pooled samples, high-resolution MS scans were collected with separate injections for positive and negative ion modes. Only data for three fatty acids (arachidonic acid, eicosapentaenoic acid, and docosahexaenoic acid) from the negative mode are presented in this study.

*Metabolomic data processing*

Metabolite peaks obtained from mass spectrometry were integrated using Skyline for small molecules (8). ﻿Full and abbreviated compound names are listed in Table S1, with abbreviated names used in figures throughout. Leucine and isoleucine, as well as butyryl-l-carnitine and isobutyryl-l-carnitine, did not separate chromatographically, so were integrated as a combined signal, referred to here as (iso)leucine and (iso)butyryl-l-carnitine, respectively. Integrated peak areas were subject to quality control, where peaks that did not meet minimum criteria (Table S2) were excluded from further analysis to ensure correct compound identification and peak quality. Only compounds detected in at least three samples and at least 2 of 3 sample replicates (excluding sea-ice samples where samples were not collected in triplicate for Sea ice_2 and Sea ice_3) were included in further analysis. As in Heal et al. (2019), for compounds that were detected or passed through quality control in only a subset of replicates, the remaining replicates were assigned a value representing an upper estimate of how large a peak could be and still remain below the detection limit (3 x peak area in blank + 100). Peak areas were then normalized using best-matched internal standard normalization to reduce variability due to ﻿changes in instrument response throughout the run (Boysen et al., 2018). As in Heal et al. (2019), a 20% improvement to the relative standard deviation of each compound in a pooled sample was used as criteria to apply normalization; compounds with a raw RSD of <10% across the raw pooled areas were not normalized. Three polyunsaturated fatty acids (arachidonic acid, eicosapentaenoic acid, and docosahexaenoic acid) that were quantified from the organic extract were not subject to best-matched internal standard normalization since concentrations were calculated directly from raw peak areas, as detailed below.

*Metabolite concentration calculations*

Absolute concentrations of compounds were calculated or estimated from peak areas for 134 metabolites using commercially available standards run in the same batch as our samples, similar to previous work (Dawson et al., 2020b; Boysen et al., 2020; Heal et al., 2020). In short, for metabolites where isotopically labeled standards were added to the samples as part of the internal standard suite (“Matched compound” in Table S3), concentrations were calculated directly (labeled “isotopologue” in quantification method of Table S1). For compounds without isotopologues, approximate concentrations were estimated by correcting for ionization efficiency and ion suppression using authentic standards mixed into water and a representative matrix by calculation of response factor (RF) and RF ratio as in previous work (Dawson et al., 2020b; Boysen et al., 2020; Heal et al., 2020), labeled “direct RF and RFratio” in quantification methods of Table S3. For glutathione, a literature value for RFratio (Boysen et al., 2018) was assumed, rather than using the calculated value which was negative, labeled “direct RF and RFratio assumed” in quantification methods of Table S1 (which contains all quantification details). Dimethylsulfoniopropionate (DMSP) may volatilize during sample processing resulting in some loss (13), but good agreement across replicates suggests that potential losses were similar across samples and that estimated concentrations presented here can be taken as a minimum value. The free concentrations of three polyunsaturated fatty acids (arachidonic acid, eicosapentaenoic acid, and docosahexaenoic acid) were calculated directly using isotopically labeled standards (Table S3) added to the samples (“isotopologue”, as above). Fatty acid data was used only to contextualize the results on certain metabolites from the aqueous extract, and are not included in analyses of “all metabolites”.

*DNA extraction, sequencing, and processing*

DNA extraction, sequencing, and sequence analysis were performed following Erazo and Bowman (2021). Briefly, DNA was extracted using the Qiagen DNeasy Power Water Kit following the manufacturer protocol with an additional heating step to aid lysis. Extracted DNA concentration was measured using the Qubit HS DNA quantification kit (Invitrogen) and quality-checked by gel electrophoresis and PCR amplification of the V3-V4 region of the 16S rRNA gene using primers ﻿515F and 806R (15) for Bacteria and Archaea and the V9 region of the 18S rRNA gene using primers 1380F and 1510R (16). High quality DNA was submitted for sequencing to the Argonne National Laboratory sequencing center for amplification and library preparation with the same primer set, followed by 2 x 151 paired-end sequencing on the Illumina Miseq platform. Reads generated from Illumina MiSeq were then demultiplexed using the ‘iu-demultiplex’ command in Illumina utils (17) quality-controlled, denoised, and merged using the dada2 package (Callahan et al., 2016a) in R. Reads were then analyzed using the ﻿paprica pipeline (Bowman and Ducklow, 2015; https://github.com/bowmanjeffs/paprica) to obtain community structure for all unique amplicon sequence variants (ASVs) by placing reads on a phylogenetic tree created from the complete 16S +23S rRNA or 18S rRNA genes from all completed genomes in the NCBI RefSeq database (20). ASVs are named according to their lowest consensus taxonomic ranking for phylogenetic placements to terminal edges (closest completed genomes, CCGs) or according to their closest relative on the phylogenetic reference tree for placements to non-terminal edges (﻿closest estimated genomes, CEGs). Distinct ASVs assigned the same taxonomic name are differentiated throughout by a number following the name (e.g. *Rhizosolenia pungens*.12 versus *Rhizosolenia pungens*.13). The paprica pipeline relies on Infernal for read alignment (21), EPA-ng for read placement (22), Gappa (23), and the PR2 (24) and RefSeq (20) databases. Reads in the 16S rRNA dataset assigned to chloroplasts were discarded.

*Statistical approaches*

Differences in community structure, both prokaryotic and eukaryotic, and metabolite composition were evaluated for each dataset separately using non-metric dimensional scaling (NMDS) (25). NMDS analyses on community structure were based on a Bray-Curtis dissimilarity matrix by Hellinger-transformed ASV data. NMDS using a Euclidean distance matrix, based on the proportional contribution of each metabolite to the quantified metabolite carbon pool (mole fraction of carbon), was used to compare samples based on targeted metabolite profiles. This approach accommodates our low sample numbers, high variable numbers, and the non-normal distribution of metabolomics data to avoid overfitting (26). Dimensionality of each NMDS was assessed with a scree plot, and probability was calculated with a Monte Carlo permutation test. Analysis of similarity (ANOSIM) (27) was used to evaluate significant differences with respect to field sample types and incubation treatments. ANOSIMS were performed with 999 permutations for nonsignificant results (*p* > 0.05) and 19999 permutations for significant results (*p* < 0.05) to obtain exact *p* values, where possible. Data transformation, standardization, NMDS, and ANOSIMS statistics were done in R using the vegan (v2.5.7) package (Oksasen et al. 2020). Metabolite contributions to the sample ordination ﻿were quantified using the envfit function from the R vegan package (Oksasen et al. 2020) with 1000 permutations to assess their correlation with the ordination (R^2^) and significance, and ﻿*p* values corrected for false discovery rate (28).

Overall degree of congruency between our prokaryotic and eukaryotic community structures was tested using a Procrustean superimposition approach (29). The Procrustes function in the R package vegan (Oksasen et al. 2020) was applied to the NMDS scores generated for each dataset. A ﻿PROcrustean Randomization TEST (PROTEST; (30)) with 19999 permutations was then used to assess the statistical significance of the Procrustean fit using the vegan package (Oksasen et al. 2020). Unique ASV correlations were performed using the Spearman’s rank correlation coefficient using the psych (v.2.1.9) package in R (Revelle 2021) with ﻿*p* values corrected for false discovery rate (28). Relative abundance data was first centered log-ratio (CLR) transformed (31) prior to correlation analyses to ensure compositional robustness in R. Correlations were calculated both using the entire sample set including field and experimental incubation samples (32 total) and with field samples only (23 total). Correlation results were visualized as a network using the igraph package in R (32).

Individual metabolite concentrations as ﻿molar carbon concentration relative to POC concentration (nmol C µmol C^-1^) were compared between incubation treatments using one-way analysis of variance (ANOVA) in R, with ﻿*p* values corrected for false discovery rate (28). Metabolites with significantly different concentrations across treatments were then clustered based on Euclidean distances and average-linkage clustering using the dist and hclust functions from the stats R package. One-way ANOVAs were also used to assess statistical differences in ancillary measurements (diversity, etc.) between samples. ﻿Post-hoc Tukey’s HSD (honestly significant difference) tests were used to explore specific significant relationships between all treatments when a significant overall effect of treatment was observed. For all statistical analyses, a probability level of ≤ 0.05 was used to determine statistical significance.

**References**

1. Guillard RRL. Culture of Phytoplankton for Feeding Marine Invertebrates. In: W.L. S, M.H. C, editors. Culture of Marine Invertebrate Animals. Boston, MA: Springer; 1975. p. 29–60.

2. Harrison PJ, Waters RE, Taylor FJR. A broad spectrum artificial seawater medium for coastal and open ocean phytoplankton. J Phycol. 1980;16:28–35.

3. Boysen AK, Heal KR, Carlson LT, Anitra E. Best-matched internal standard normalization in liquid chromatography-mass spectrometry environmental metabolomics. Anal Chem. 2018;90:﻿1363–69.

4. Heal KR, Durham BP, Boysen AK, Carlson LT, Qin W, Ribalet F, et al. Marine Community Metabolomes carry fingerprints of phytoplankton community composition. mSystems. 2021;6:1–19

5. Krembs C, Eicken H, Deming JW. Exopolymer alteration of physical properties of sea ice and implications for ice habitability and biogeochemistry in a warmer Arctic. Proc Natl Acad Sci U S A. 2011;108:3653–8.

6. Knap A., Michaels A., Close A., Ducklow H., Dickson A. Protocols for the Joint Global Ocean Flux Study ( JGOFS ) Core Measurements. UNESCO. 1996;

7. N.A. W. Fluorometric analysis of chlorophyll a in the presence of chlorophyll b and pheopigments. Limnol Oceanogr. 1994;39:1985–92.

8. MacLean B, Tomazela DM, Shulman N, Chambers M, Finney GL, Frewen B, et al. Skyline: An open source document editor for creating and analyzing targeted proteomics experiments. Bioinformatics. 2010;26:966–8.

9. Heal KR, Kellogg NA, Carlson LT, Lionheart RM, Ingalls AE. Metabolic Consequences of Cobalamin Scarcity in the Diatom Thalassiosira pseudonana as Revealed Through Metabolomics. Protist [Internet]. 2019;170:328–48.

10. Boysen AK, Carlson LT, Durham BP, Groussman RD, Aylward FO, Ribalet F, et al. Particulate metabolites and transcripts reflect diel oscillations of microbial activity in the surface ocean. mSystems. 2021;6:1–18.

11. Dawson HM, Heal KR, Torstensson A, Carlson LT, Ingalls AE, Young JN. Large diversity in nitrogen- and sulfur-containing compatible solute profiles in polar and temperate diatoms. Integr Comp Biol. 2020;60:1401–13.

12. Dawson HM, Heal KR, Boysen AK, Carlson LT, Ingalls AE, Young JN. Potential of temperature- and salinity-driven shifts in diatom compatible solute concentrations to impact biogeochemical cycling within sea ice. Elem Sci Anth. 2020;8:1–17 .

13. Spielmeyer A, Pohnert G. Direct quantification of dimethylsulfoniopropionate (DMSP) with hydrophilic interaction liquid chromatography/mass spectrometry. J Chromatogr B, Anal Technol Biomed life Sci. 2010 Dec;878:3238–42.

14. Erazo NG, Bowman JS. Sensitivity of the mangrove-estuarine microbial community to aquaculture effluent. iScience [Internet]. 2021;24:102204.

15. Walters W, Hyde ER, Berg-lyons D, Ackermann G, Humphrey G, Parada A, et al. Transcribed Spacer Marker Gene Primers for Microbial Community Surveys. mSystems. 2015;1:e0009–15.

16. Amaral-Zettler LA, McCliment EA, Ducklow HW, Huse SM. A method for studying protistan diversity using massively parallel sequencing of V9 hypervariable regions of small-subunit ribosomal RNA Genes. PLoS One. 2009;4:1–9.

17. Eren AM, Maignien L, Sul WJ, Murphy LG, Grim SL, Morrison HG, et al. Oligotyping: Differentiating between closely related microbial taxa using 16S rRNA gene data. Methods Ecol Evol. 2013;4:1111–9.

18. Callahan BJ, McMurdie PJ, Rosen MJ, Han AW, Johnson AJA, Holmes SP. DADA2: High-resolution sample inference from Illumina amplicon data. Nat Methods. 2016;13:581–3.

19. Bowman JS, Ducklow HW. Microbial communities can be described by metabolic structure: A general framework and application to a seasonally variable, depth-stratified microbial community from the coastal West Antarctic Peninsula. PLoS One. 2015;10:1–18.

20. Haft DH, DiCuccio M, Badretdin A, Brover V, Chetvernin V, O’Neill K, et al. RefSeq: An update on prokaryotic genome annotation and curation. Nucleic Acids Res. 2018;46:D851–60.

21. Nawrocki EP, Eddy SR. Infernal 1.1: 100-fold faster RNA homology searches. Bioinformatics. 2013;29:2933–5.

22. Barbera P, Kozlov AM, Czech L, Morel B, Darriba D, Flouri T, et al. EPA-ng: Massively Parallel Evolutionary Placement of Genetic Sequences. Syst Biol. 2019;68:365–9.

23. Czech L, Barbera P, Stamatakis A. Genesis and Gappa: Processing, analyzing and visualizing phylogenetic (placement) data. Bioinformatics. 2020;36:3263–5.

24. Guillou L, Bachar D, Audic S, Bass D, Berney C, Bittner L, et al. The Protist Ribosomal Reference database (PR2): A catalog of unicellular eukaryote Small Sub-Unit rRNA sequences with curated taxonomy. Nucleic Acids Res. 2013;41:597–604.

25. Kruskal J, Wish M. Multidimensional Scaling [Internet]. Thousand Oaks, California; 1978. Available from: https://methods.sagepub.com/book/multidimensional-scaling

26. Saccenti E, Hoefsloot HCJ, Smilde AK, Westerhuis JA, Hendriks MMWB. Reflections on univariate and multivariate analysis of metabolomics data. Metabolomics. 2014;10:361–74.

27. Clarke KR. Non‐parametric multivariate analyses of changes in community structure. Aust J Ecol. 1993;18:117–43.

28. Benjamini Y, Hochberg Y. Controlling the False Discovery Rate: a Practical and Powerful Approach to Multiple Testing. J R Stat Soc. 1995;57:289–300.

29. Gower JC. Statistical methods of comparing different multivariate analyses of the same data. In: FR H, DG K, P T, editors. Mathematics in the archaeological and historical science. Edinburgh: Edinburgh University Press; 1971. p. 138–49.

30. Jackson DA. PROTEST: A PROcrustean Randomization TEST of community environment concordance. Écoscience. 1995;2:297–303.

31. Aitchison J, Society S, Methodological SB. The Statistical Analysis of Compositional Data The Statistical Analysis of Compositional Data. 1982;44:139–77.

32. Csardi G, Nepusz T. The igraph software package for complex network research. InterJournal [Internet]. 2006;Complex Sy:1695. Available from: https://igraph.org

33. Oksanen, F.J., et al. (2017) Vegan: Community Ecology Package. R package Version 2.5-7. https://CRAN.R-project.org/package=vegan

34. Revelle W (2022). psych: Procedures for Psychological, Psychometric, and Personality Research. Northwestern University, Evanston, Illinois. R package version 2.1.9, https://CRAN.R-project.org/package=psych.
