# Supplementary material for: Microbial metabolomic responses to changes in temperature and salinity along the western Antarctic Peninsula
Source: ISME J. 2023 Sep 15;17(11):2035–46. doi: 10.1038/s41396-023-01475-0 (PMC10579395; doi:10.1038/s41396-023-01475-0)
Supplement: Supplementary file 2 — Supplemental Figures [file 41396_2023_1475_MOESM2_ESM.pdf]

**Supplemental figures**

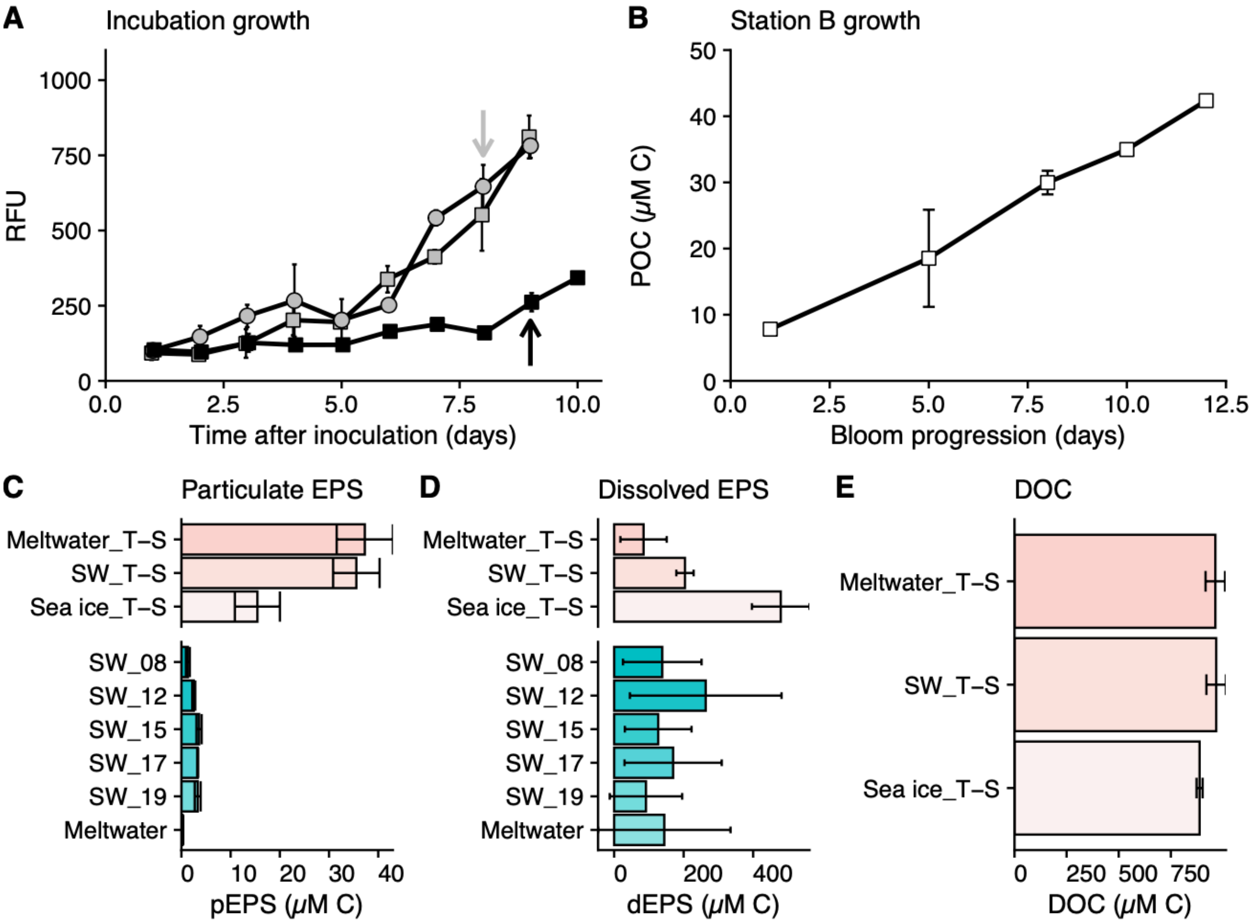


Figure S1. Ancillary data from incubation and field samples. a) Growth curves of incubation treatments under the three experimental conditions, with symbols as in Figure 1a. Treatments grown at meltwater (grey circles) and seawater (grey squares) conditions were harvested at grey arrow and treatments grown at sea ice conditions were harvested at black arrow. RFU = relative fluorescence units, error bars are standard deviation ($n$ = 3 for all time points). b) Growth curve of Station B seawater samples (SW_08 – SW_19). POC = particulate organic carbon, error bars are standard deviation ($n$ = 3 for all time points). c) Concentration of particulate extracellular polysaccharides (pEPS) in incubation and field samples, shown in terms of µM carbon equivalents. Error bars are standard deviation ($n$ = 3 for all). The x-axis break separates incubation treatment samples on the left and field samples on the right. d) Concentration of dissolved extracellular polysaccharides (dEPS) in incubation and field samples, shown in terms of µM carbon equivalents. Error bars are standard deviation ($n$ = 2 for SW_T-S, Sea ice_T-S, and Meltwater, $n$ = 3 for rest). The x-axis break separates incubation treatment samples on the left and field samples on the right. e) Concentration of dissolved organic carbon (DOC) in incubation and field samples, shown in terms of µM carbon equivalents. Error bars are standard deviation of the mean ($n$ = 3 for the meltwater treatment, $n$ = 2 for the seawater and sea-ice treatments, and $n$ = 1 for the seawater and meltwater field samples). Full data for panels c – e are available in Supplementary Table S4. Note that we do not have ancillary chemical measurements to pair with the sea-ice samples.

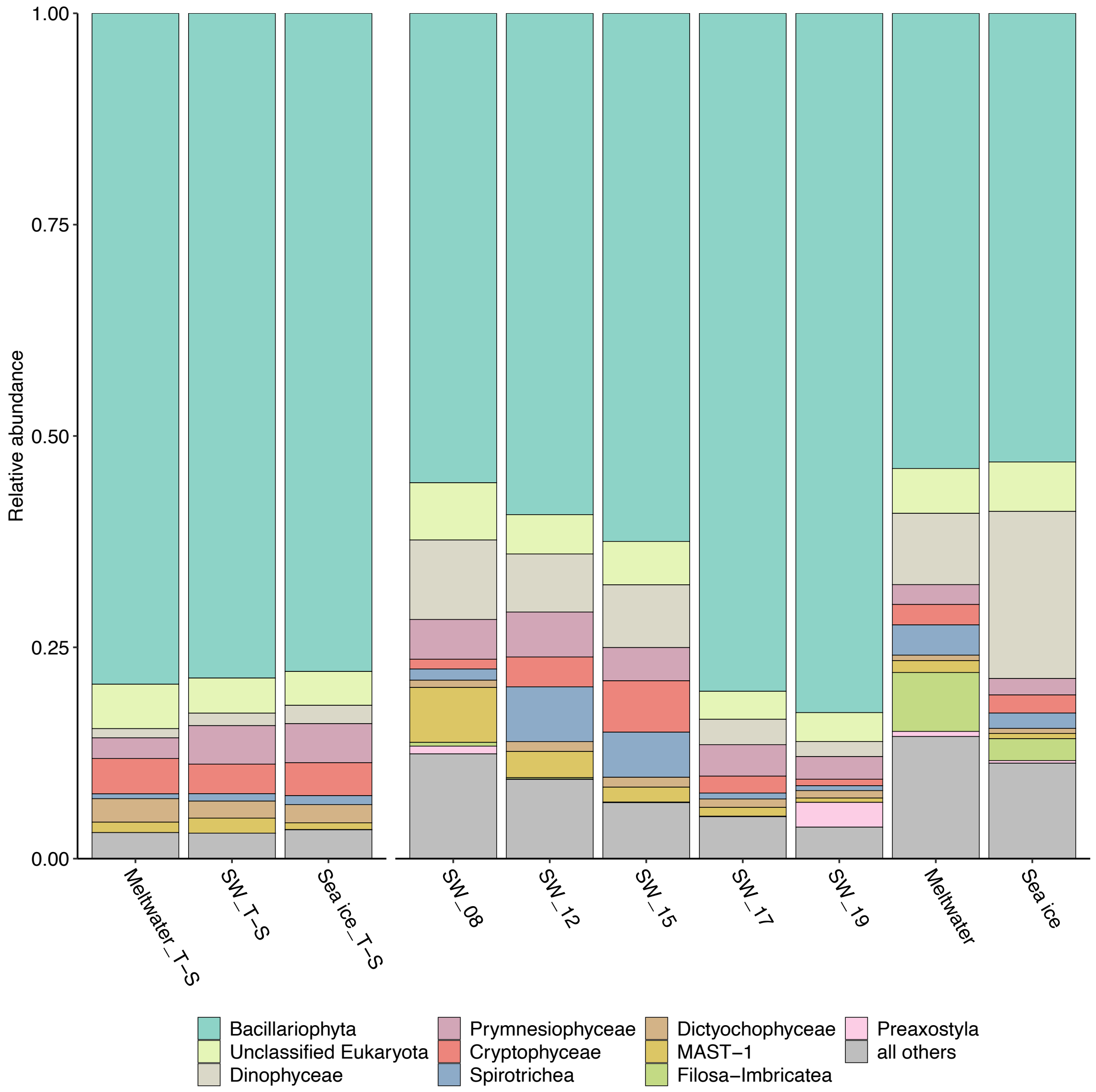


Figure S2. Relative abundance of eukaryotic taxa in field and incubation samples at the class level. Average of triplicates is shown, except for sea-ice core where $n = 5$. The most abundant 10 classes across the sample set for each sample are shown, with "all others" containing the sum of the remaining classes. The x-axis break separates incubation treatment samples on the left and field samples on the right. Full data available in Table S5.

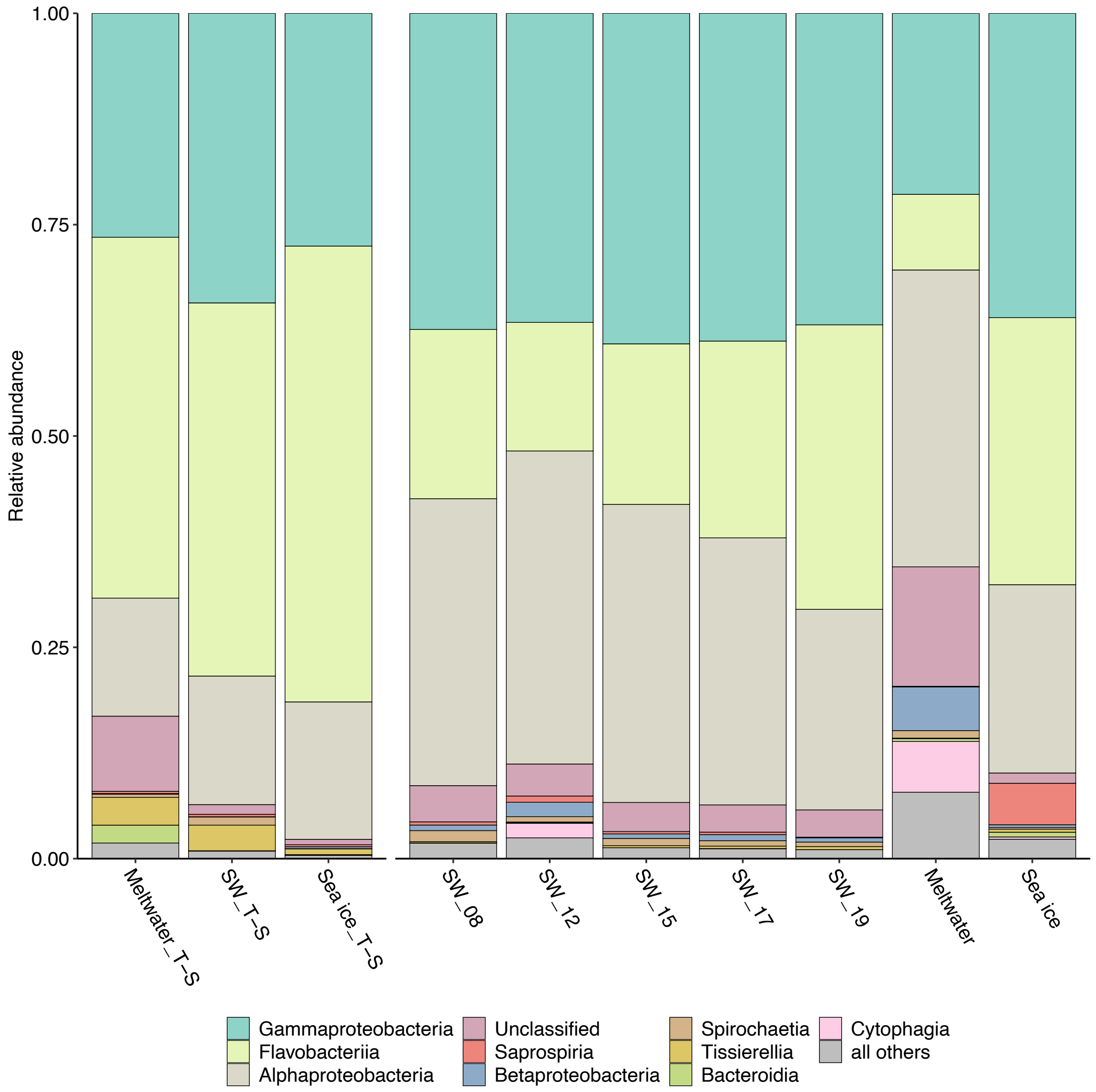


Figure S3. Relative abundance of prokaryotic taxa in field and incubation samples at the class level. Average of triplicates is shown, except for sea-ice core where *n* = 5. The most abundant 10 classes across the sample set for each sample are shown, with "all others" containing the sum of the remaining classes. The x-axis break separates incubation treatment samples on the left and field samples on the right. Full data available in Table S6.

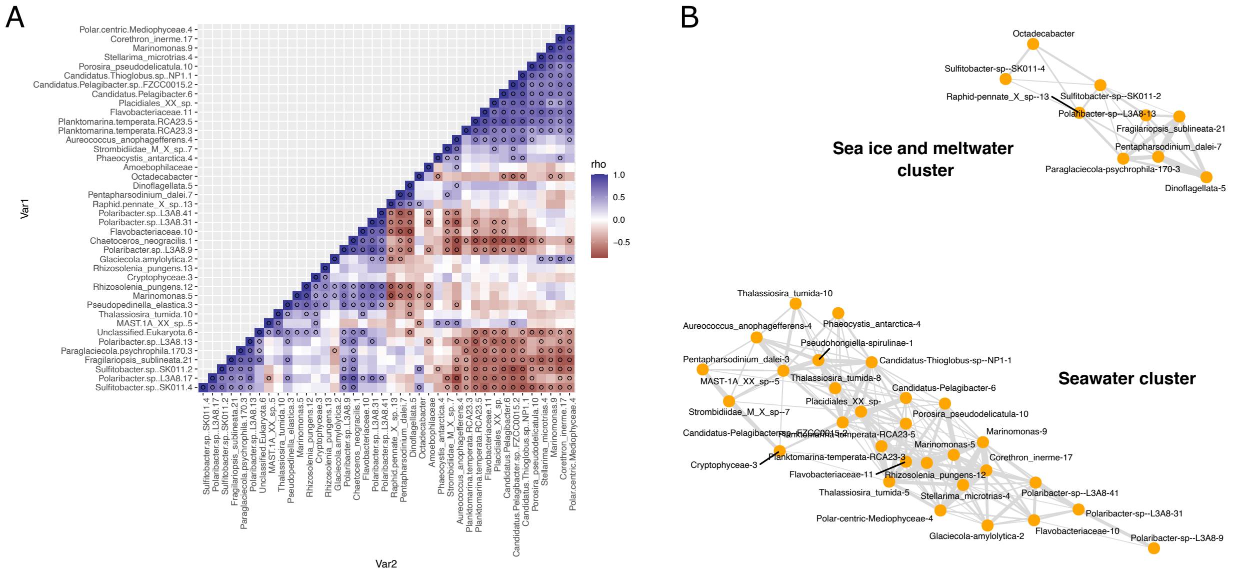


Figure S4. Correlation patterns between most abundant eukaryotic and prokaryotic ASVs. a) Correlations for the top 20 prokaryotic and eukaryotic ASVs across entire sample set (incubation and field samples, 32 in total) based on the Spearman correlation of centered log-ratio-transformed data. Significant correlations (fdr-corrected *p* value < 0.05) are denoted with black circles. b) Network visualization of significant ($p < 0.05$) positive correlations between ASVs across field samples only. Each ASV is depicted as a node (as labeled in Figure 3). The width of edges is proportional to the strength of the correlation (Spearman correlation coefficient). Correlation and significance data for all samples and for field samples only are available in Tables S9 and S10, respectively.

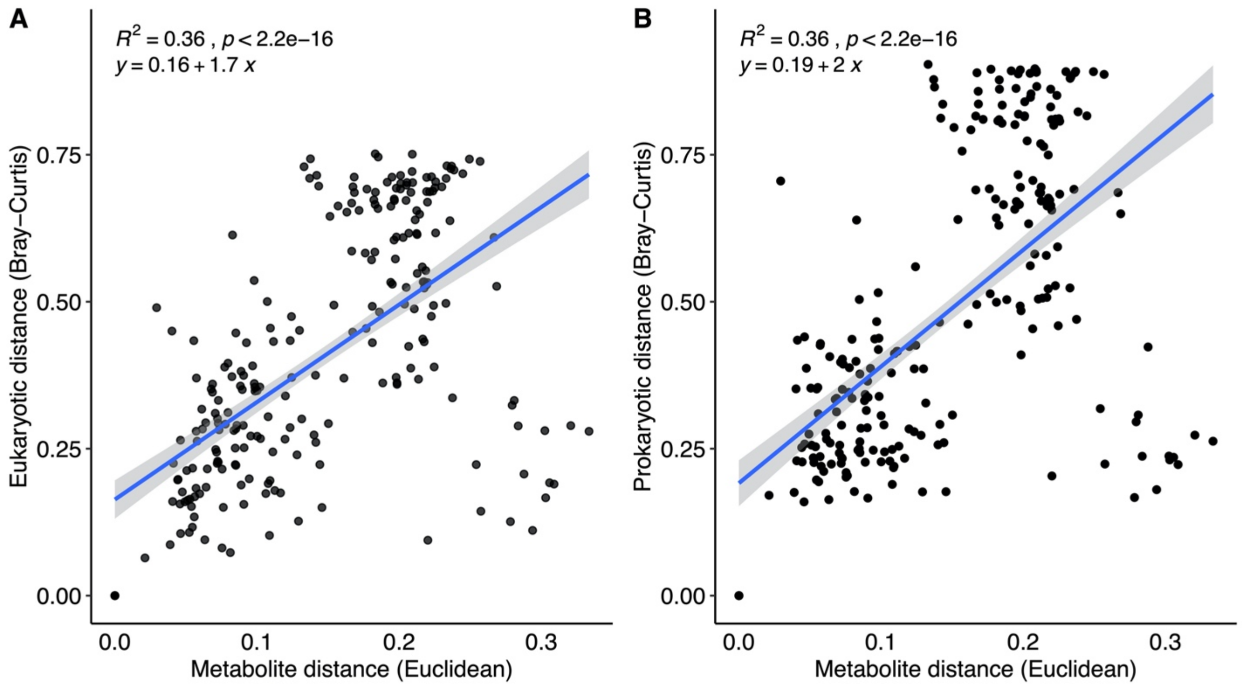


Figure S5. Sample similarity in metabolite space compared to community structure space. Pairwise sample distances in metabolite space (Euclidean distance) compared to sample distances in eukaryotic (A) and prokaryotic (B) space (Bray-Curtis dissimilarity) in field samples from sea ice, meltwater, and seawater. Incubation treatment samples are not included. Linear regression statistics are provided on the plot. The shaded area represents a pointwise 95% confidence interval of the fitted values. Full data available in Supplementary Table S13.

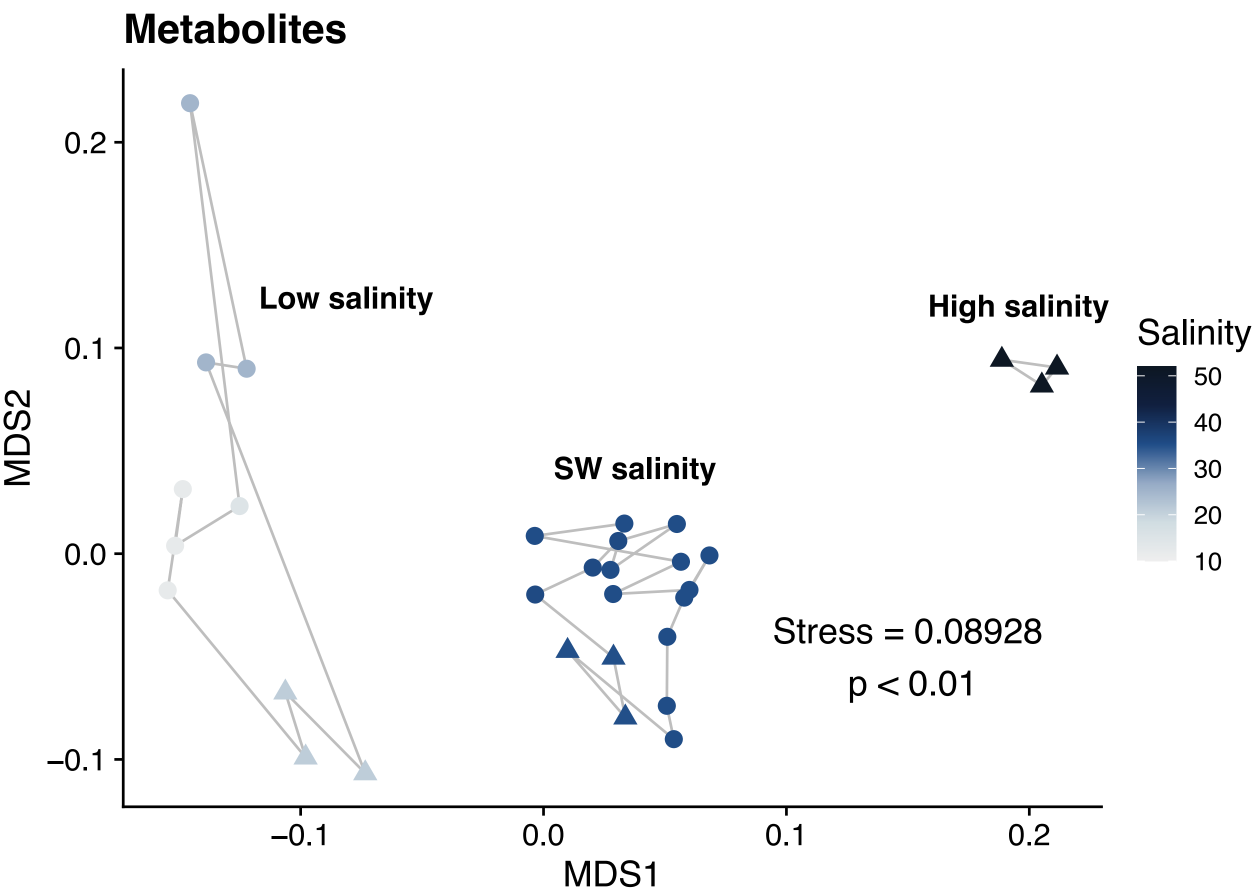


Figure S6. Impact of salinity status on metabolite composition. NMDS ordination comparing the mole fraction of carbon metabolite composition of each sample (as shown in Figure 4d) with colors showing the measured sample salinity conditions in ppt. Samples with similar salinity status (from left to right as labeled on plot: Low salinity = Meltwater_T-S, Meltwater, and Sea ice; SW salinity = Seawater_T-S and all field seawater samples; and High salinity = Sea ice_T-S;) are shown connected, with incubation treatment samples in triangles and field samples in circles. Full salinity data can be found in Table 1. Full metabolomics data can be found in Table S11.

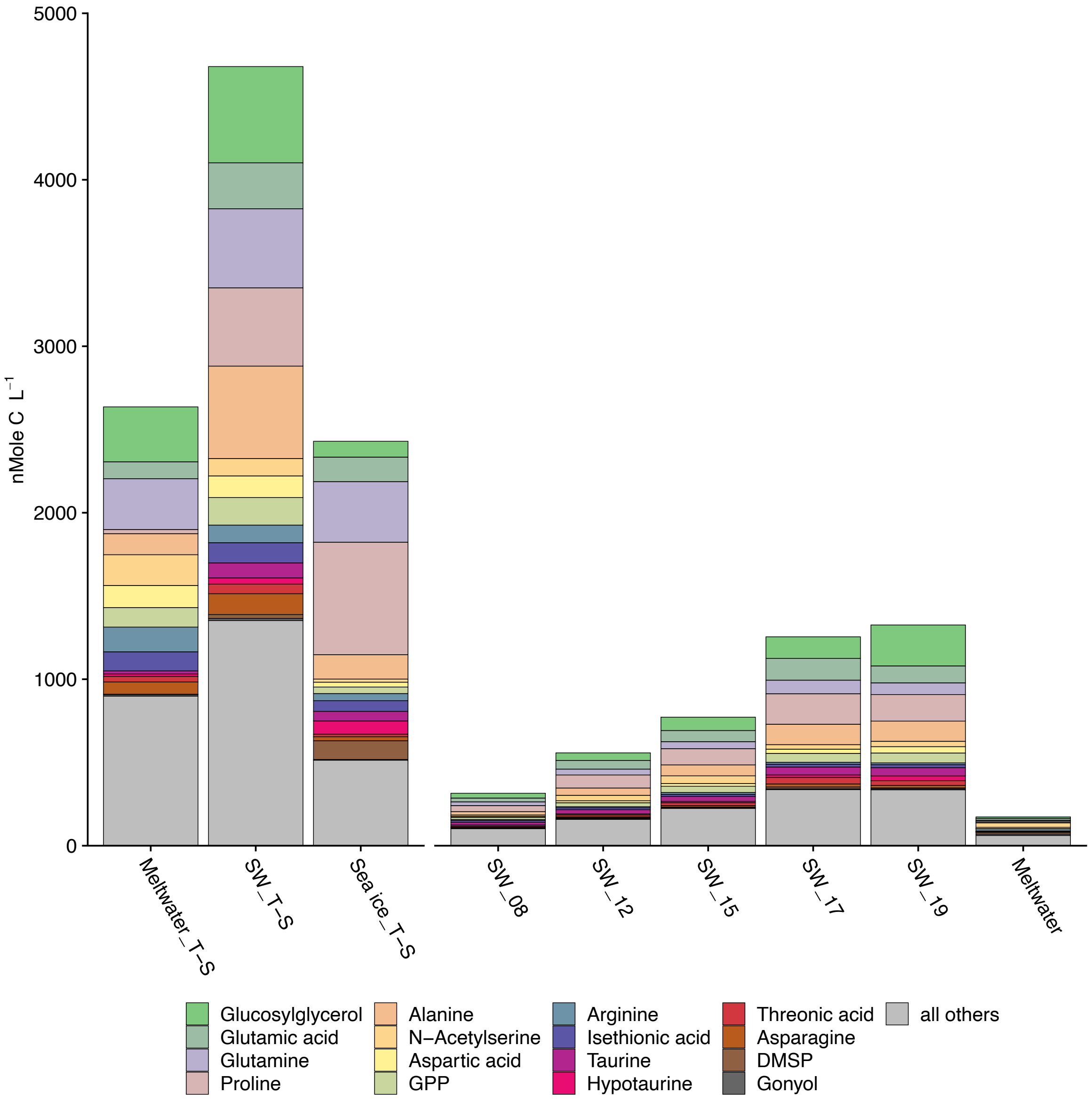


Figure S7. Estimated particulate concentrations (nmol metabolite C per L) of metabolites across incubation and field samples. Average of triplicates is shown. The most abundant 16 molecules for each sample are shown, with "all others" containing the sum of the remaining quantified metabolites (118). Note that we do not have exact dilution factor data to pair with the sea-ice samples, so they are excluded from this figure. The x-axis break separates incubation treatment samples on the left and field samples on the right. Full data available in Table S11.

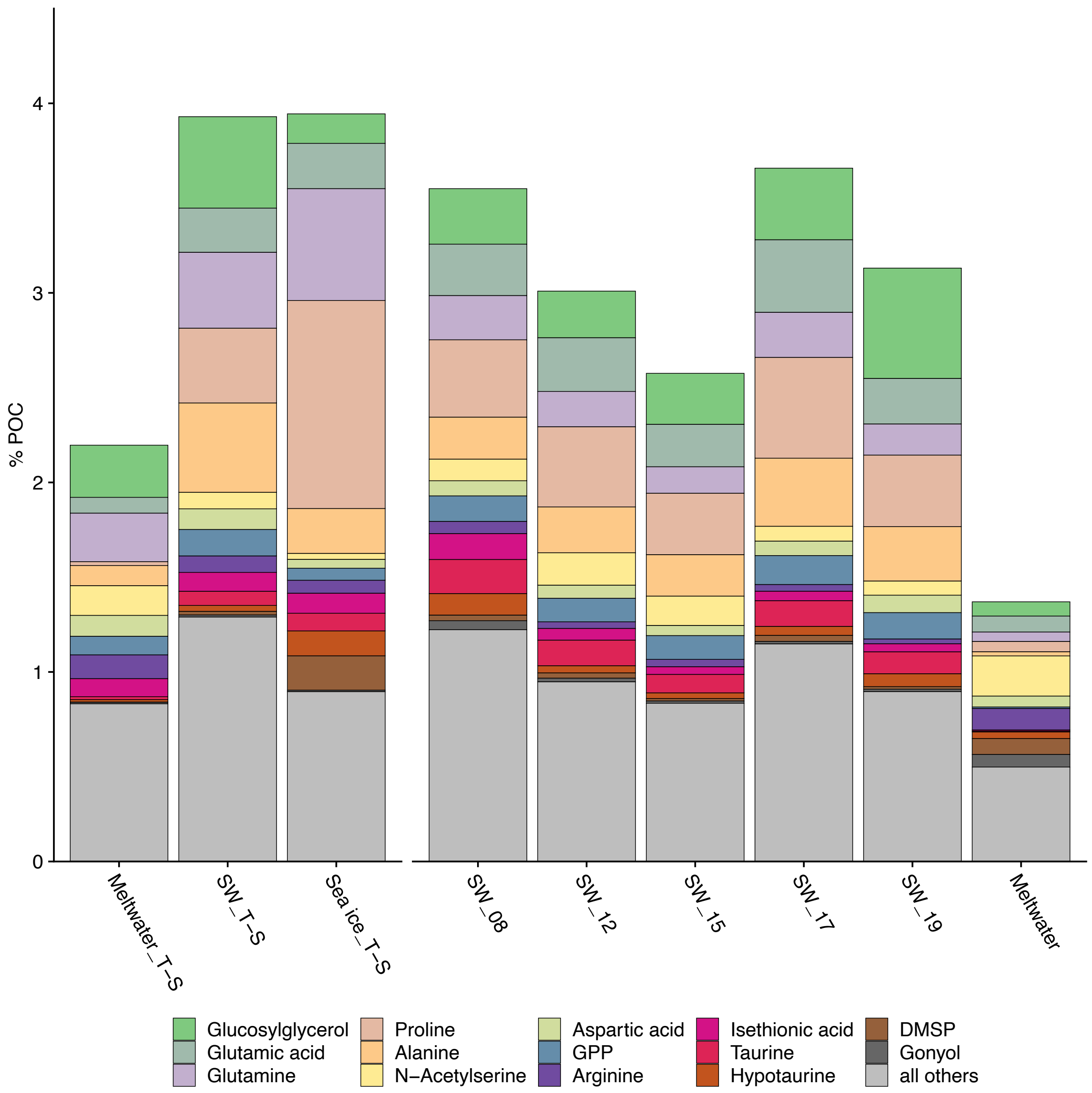


Figure S8. Total quantified metabolite concentration as the percentage of particulate organic carbon (POC). Average of triplicates is shown. The most abundant 15 molecules for each sample are shown, with "all others" containing the sum of the rest of the metabolites quantified (119). Note that we do not have POC or PN to pair with the sea-ice samples. The x-axis break separates incubation treatment samples on the left and field samples on the right. Full data available in Table S14.

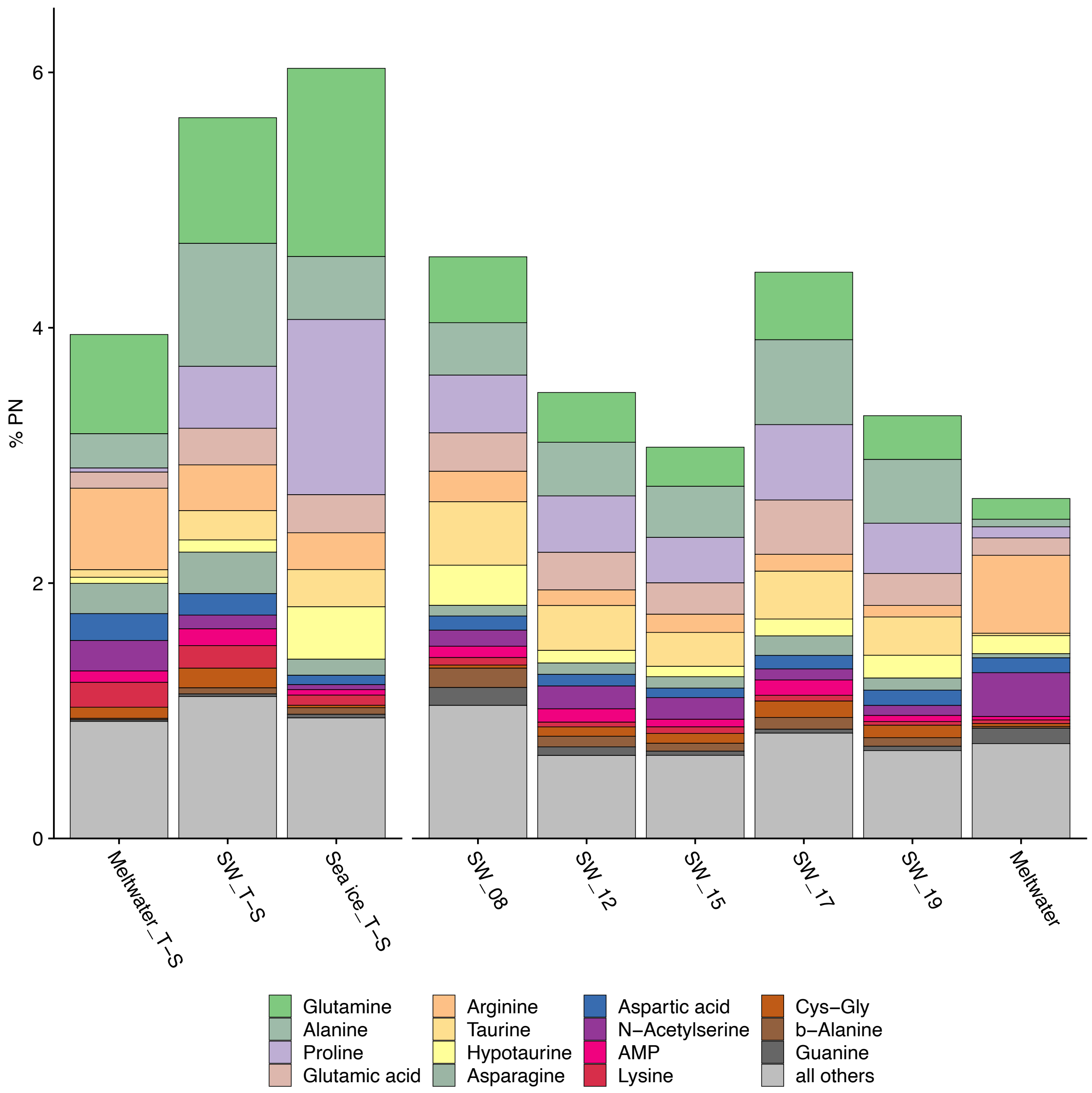


Figure S9. Total quantified metabolite concentration as the percentage of particulate organic nitrogen (PN). Average of triplicates is shown. The most abundant 15 molecules for each sample are shown, with "all others" containing the sum of the rest of the metabolites quantified (119). Note that we do not have particulate carbon or nitrogen paired with the sea-ice samples. The x-axis break separates incubation treatment samples on the left and field samples on the right. Full data available in Supplementary Table S14.

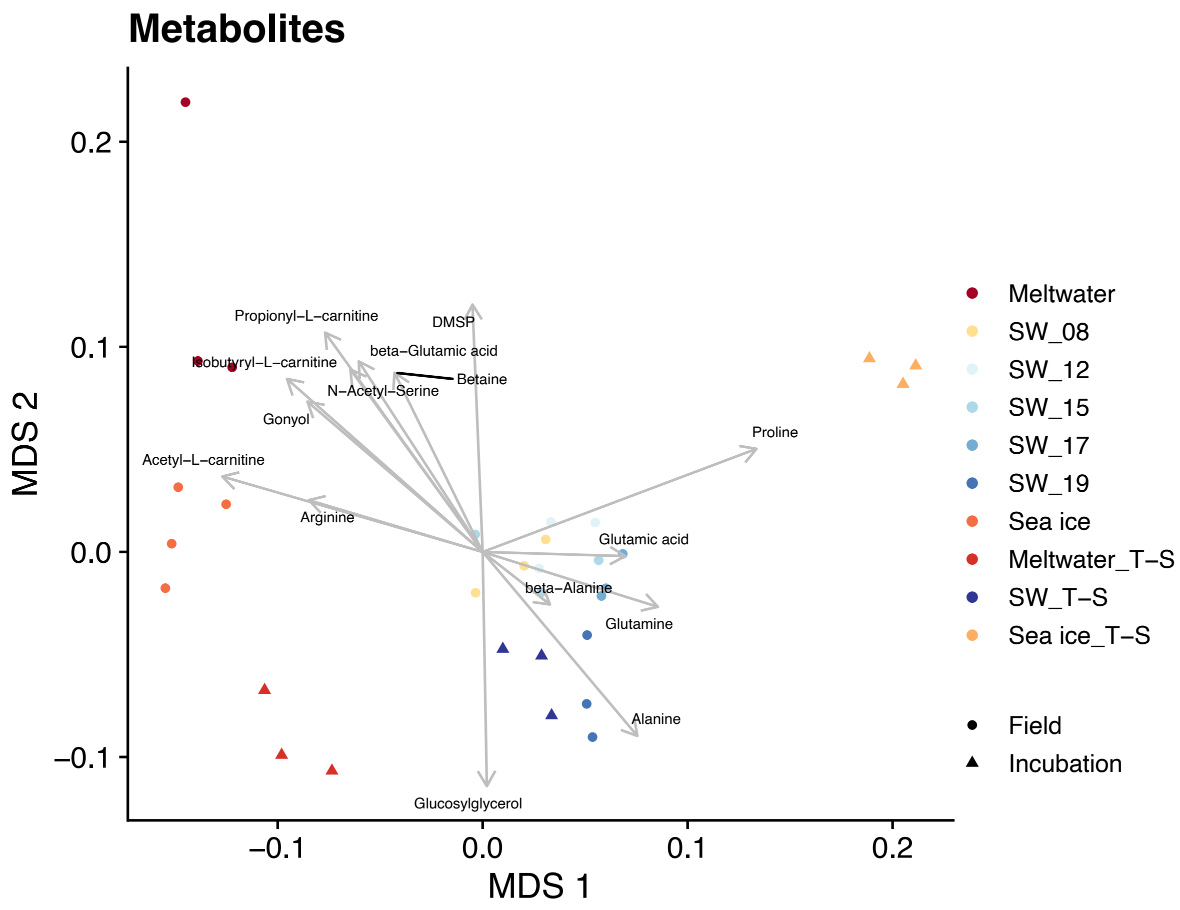


Figure S10. Metabolite contributions to the sample ordination. NMDS ordination comparing the mole fraction of carbon metabolite composition of each sample (as shown in Figure 4d) with overlain vectors showing metabolite “loadings” (i.e., variable weights) on each derived axis from the NMDS, calculated using the function envfit() from the vegan package. Vector lengths are scaled by their correlation (square root of R2) so that “weak” predictors have shorter arrows than “strong” predictors. Vector directions in ordination space point toward the metabolites that change most rapidly and to which metabolite they have maximal correlations with the ordination configuration (i.e., as you travel along each vector, the samples generally increase with respect to the proportional abundance of that metabolite). Significance determined using a permutation test, with $p$ values corrected for false discovery rate ($q$ value). For ease of visualization, metabolite vectors are only shown for those with statistically significant loadings on the first two NMDS axes ($q < 0.05$) that are highlighted in the main text. Full significance and correlation results for all metabolites are detailed in Table S15.

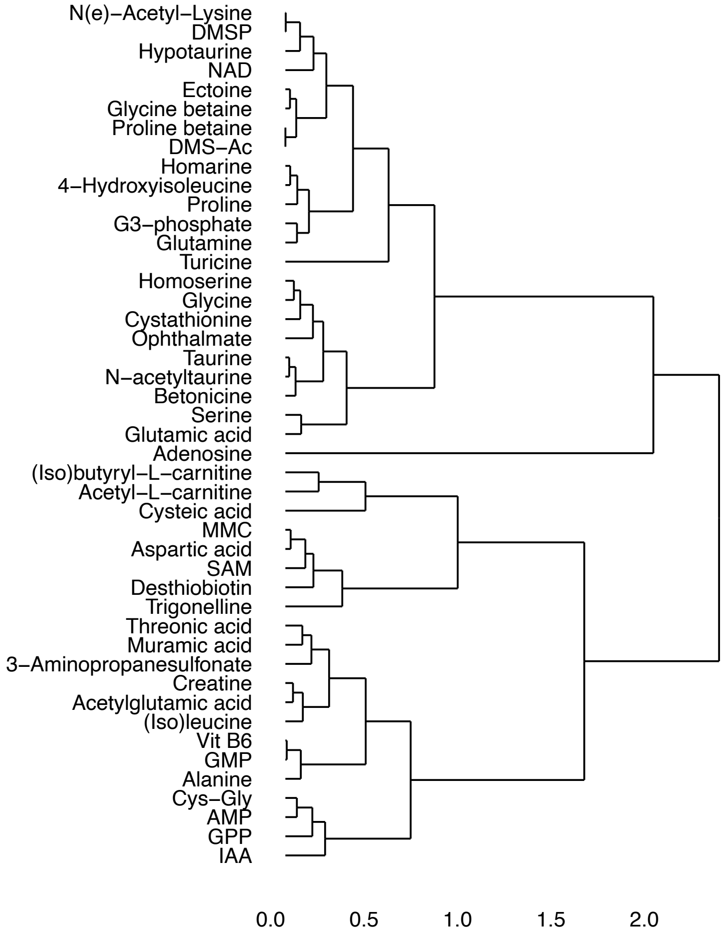


Figure S11. Patterns of metabolite response to temperature and salinity change in incubation experiments. Dendrogram of metabolites that were significantly different ($p < 0.05$) with treatment, as determined by false discovery rate-corrected $p$ values from one-way ANOVAs (detailed in Table S13). Metabolites are clustered using average linkage clustering on a Euclidean distance matrix.

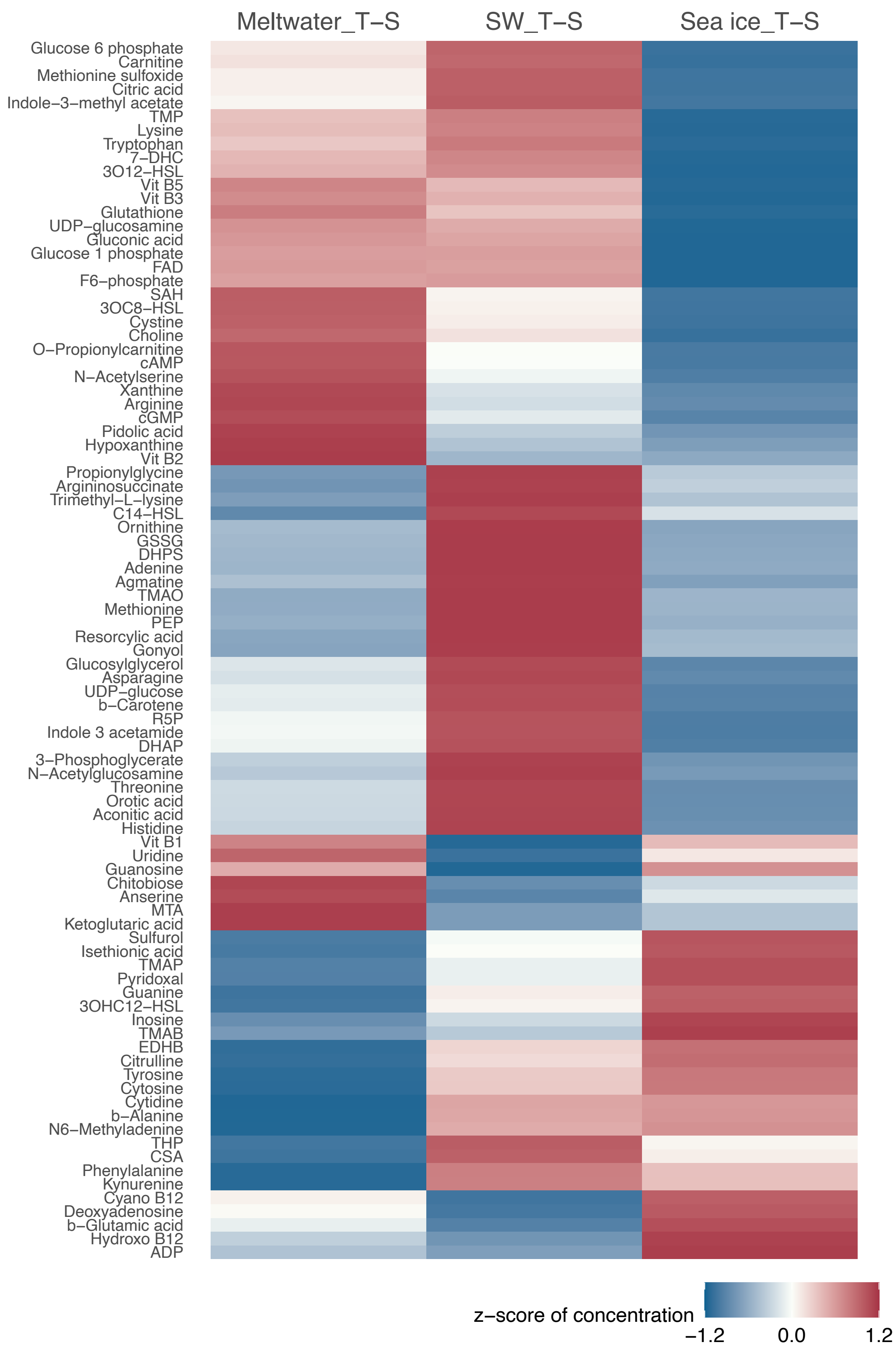
Meltwater_T−S
SW_T−S
Sea ice_T−S
Glucose 6 phosphate
Carnitine
Methionine sulfoxide
Citric acid
Indole−3−methyl acetate
TMP
Lysine
Tryptophan
7−DHC
3O12−HSL
Vit B5
Vit B3
Glutathione
UDP−glucosamine
Gluconic acid
Glucose 1 phosphate
FAD
F6−phosphate
SAH
3OC8−HSL
Cystine
Choline
O−Propionylcarnitine
cAMP
N−Acetylserine
Xanthine
Arginine
cGMP
Pidolic acid
Hypoxanthine
Vit B2
Propionylglycine
Argininosuccinate
Trimethyl−L−lysine
C14−HSL
Ornithine
GSSG
DHPS
Adenine
Agmatine
TMAO
Methionine
PEP
Resorcylic acid
Gonyol
Glucosylglycerol
Asparagine
UDP−glucose
b−Carotene
R5P
Indole 3 acetamide
DHAP
3−Phosphoglycerate
N−Acetylglucosamine
Threonine
Orotic acid
Aconitic acid
Histidine
Vit B1
Uridine
Guanosine
Chitobiose
Anserine
MTA
Ketoglutaric acid
Sulfurol
Isethionic acid
TMAP
Pyridoxal
Guanine
3OHC12−HSL
Inosine
TMAB
EDHB
Citrulline
Tyrosine
Cytosine
Cytidine
b−Alanine
N6−Methyladenine
THP
CSA
Phenylalanine
Kynurenine
Cyano B12
Deoxyadenosine
b−Glutamic acid
Hydroxo B12
ADP
z−score of concentration
−1.2
0.0
1.2

Figure S12. Particulate metabolite responses to temperature and salinity change during the incubation experiments. Heat map showing color-scaled z-score standardized concentrations of metabolites (nmol metabolite C $\mu$mol C$^{-1}$), arranged by average linkage hierarchical clustering of Euclidean distance. Compounds shown here were not significantly different ($p > 0.05$) with treatment, as determined by false discovery rate-corrected $p$ values from one-way ANOVAs (as detailed in Table S16).

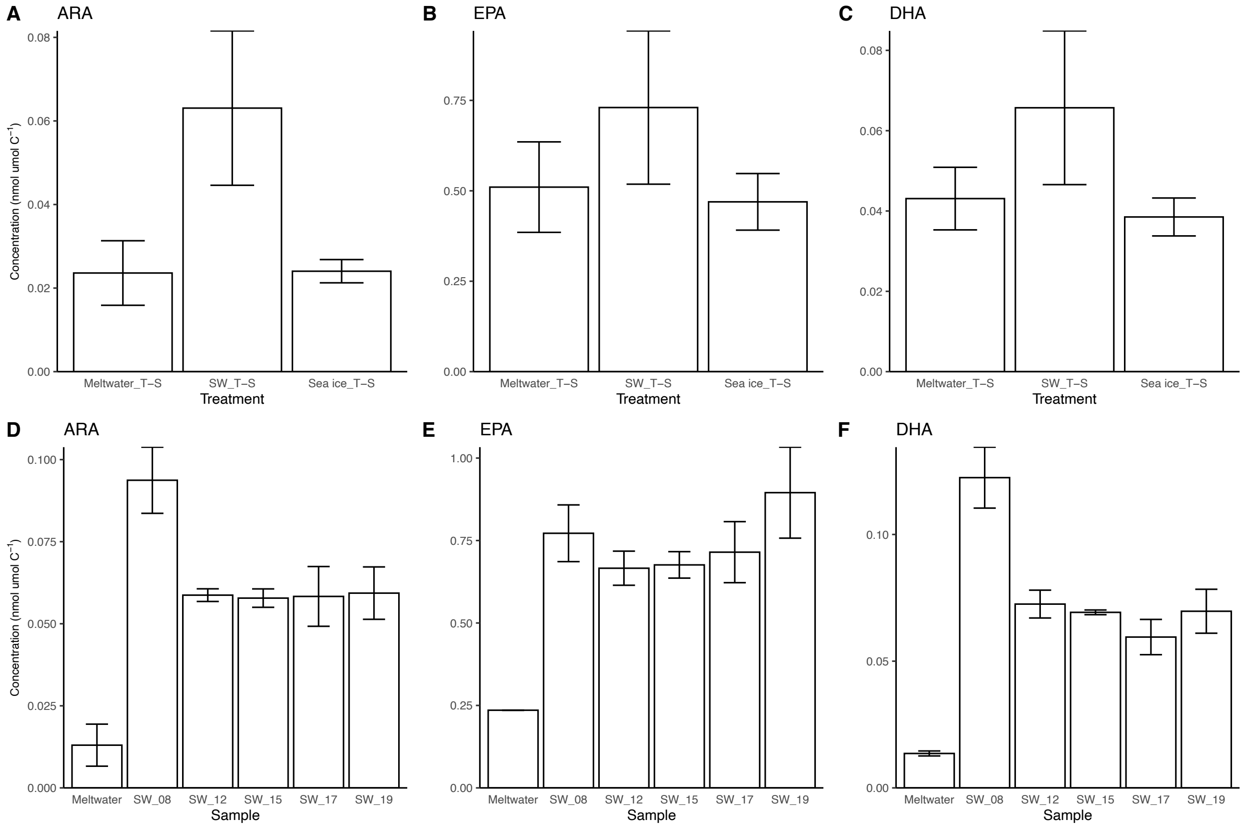


Figure S13. Select fatty acid concentrations in field and incubation samples. Particulate concentration (nmol metabolite $\mu$mol $C^{-1}$) of free fatty acids in the incubations grouped by treatment (top row) and field (bottom row) for A) and D) arachidonic acid (ARA), B) and E) Eicosapentaenoic acid (EPA), and C) and F) Docosahexaenoic acid (DHA). Error bars represent standard deviation of the mean ($n$ = 2 for Meltwater_T-S, Meltwater, and SW_15, $n$ = 3 for rest). Full data available in Table S18.

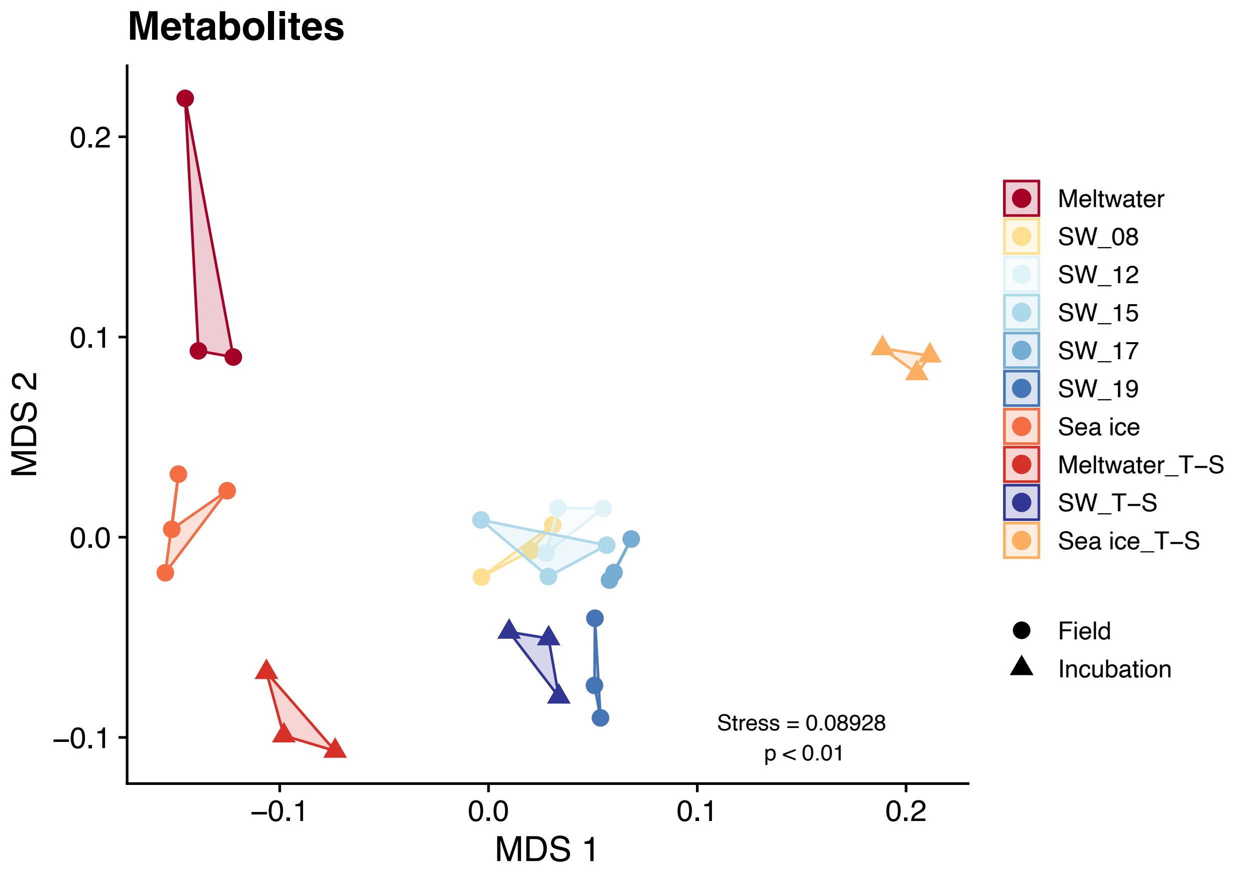


Figure S14. Multidimensional structure of metabolite composition in incubation and field samples following the removal of vitamins in f/2 amendments (Cyano B12 and Vitamin B1). Non-metric dimensional scaling (NMDS) ordination, using Euclidean distance, comparing the metabolite composition of each sample. Metabolite concentrations are scaled to mole fraction of carbon. Colors indicate sample type. Full data for metabolites are provided in Table S11. Full ANOSIM statistics for differences between groups are available in Table S19.
